# Identification of Endothelial Proteins in Plasma Associated With Cardiovascular Risk Factors

**DOI:** 10.1161/ATVBAHA.121.316779

**Published:** 2021-10-28

**Authors:** Maria J. Iglesias, Larissa D. Kruse, Laura Sanchez-Rivera, Linnea Enge, Philip Dusart, Mun-Gwan Hong, Mathias Uhlén, Thomas Renné, Jochen M. Schwenk, Göran Bergstrom, Jacob Odeberg, Lynn M. Butler

**Affiliations:** Science for Life Laboratory, Department of Protein Science, CBH, KTH Royal Institute of Technology, Stockholm, Sweden (M.J.I., L.D.K., L.S.-R., L.E., P.D., M.G.H., M.U., J.M.S., J.O., L.M.B.).; Division of Internal Medicine, University Hospital of North Norway, Tromsø (M.J.I., J.O.).; Institute for Clinical Chemistry and Laboratory Medicine, University Medical Centre Hamburg-Eppendorf, Germany (T.R.).; Irish Centre for Vascular Biology, School of Pharmacy and Biomolecular Sciences, Royal College of Surgeons in Ireland, Dublin, Ireland (T.R.).; Centre for Thrombosis and Hemostasis (CTH), Johannes Gutenberg University Medical Center, Mainz, Germany (T.R.).; Institute of Medicine, Sahlgrenska Academy at the University of Gothenburg, Sweden (G.B.).; Department of Clinical Medicine, The Arctic University of Norway, Tromsø (J.O., L.M.B.).; Coagulation Unit, Department of Hematology (J.O.), Karolinska University Hospital, Stockholm, Sweden.; Clinical Chemistry, Karolinska University Laboratory (L.M.B.), Karolinska University Hospital, Stockholm, Sweden.; Clinical Chemistry and Blood Coagulation Research, Department of Molecular Medicine and Surgery, Karolinska Institute, Stockholm, Sweden (L.M.B.).

**Keywords:** cardiovascular diseases, endothelium, hypertension, risk factors, smoking

## Abstract

**Objective:**

Endothelial cell (EC) dysfunction is a well-established response to cardiovascular disease risk factors, such as smoking and obesity. Risk factor exposure can modify EC signaling and behavior, leading to arterial and venous disease development. Here, we aimed to identify biomarker panels for the assessment of EC dysfunction, which could be useful for risk stratification or to monitor treatment response.

**Approach and Results:**

We used affinity proteomics to identify EC proteins circulating in plasma that were associated with cardiovascular disease risk factor exposure. Two hundred sixteen proteins, which we previously predicted to be EC-enriched across vascular beds, were measured in plasma samples (N=1005) from the population-based SCAPIS (Swedish Cardiopulmonary Bioimage Study) pilot. Thirty-eight of these proteins were associated with body mass index, total cholesterol, low-density lipoprotein, smoking, hypertension, or diabetes. Sex-specific analysis revealed that associations predominantly observed in female- or male-only samples were most frequently with the risk factors body mass index, or total cholesterol and smoking, respectively. We show a relationship between individual cardiovascular disease risk, calculated with the Framingham risk score, and the corresponding biomarker profiles.

**Conclusions:**

EC proteins in plasma could reflect vascular health status.

## Abstract 2

Supplemental Digital Content is available in the text.

**Highlights**

- Affinity proteomics were used to identify endothelial proteins in plasma that were associated with cardiovascular disease risk factor exposure.
- Plasma levels of 38 endothelial proteins were associated with body mass index, total cholesterol, low-density lipoprotein, smoking, hypertension, or diabetes.
- Individual cardiovascular disease risk, calculated with the Framingham risk score, was associated with the endothelial protein plasma profile.
- endothelial cell proteins in plasma could potentially be used to monitor vascular health status.

Cardiovascular disease (CVD) is the leading cause of death, killing ≈17.9 million people each year, globally (World Health Organization, 2019). Endothelial cells (EC), which line the inside of all blood vessels, are key for vascular health as they regulate hemostasis, provide an antithrombotic surface, control inflammation, vascular tone, angiogenesis, and the transport of molecules and nutrients to and from the bloodstream.^1,2^ Disruption in this normal function is termed EC dysfunction, which is linked to thrombosis formation, uncontrolled leukocyte recruitment and platelet activation, inappropriate vasoconstriction, and impaired recovery from injury.^3^ Exposure to CVD risk factors, such as smoking, obesity, hypertension, modified blood lipid profile, or diabetes can induce such changes, through the effects of chronic inflammation, oxidative stress, local hypoxia, and disruption in laminar flow.^4–8^ These changes can predispose to arterial^9,10^ and venous^11,12^ CVD development, whereas interventions that improve EC function can lessen these effects.^3,13–15^ Indeed, some successful therapies for CVD have transpired to act, at least partly, through EC protective effects (eg, angiotensin-converting enzyme inhibitors and statins), although they were not originally designed to function via this mechanism.^15,16^

Vasoreactivity has been used to assess EC dysfunction, but it can be invasive and time-consuming to measure, and its prognostic value is debated.^13^ Plasma levels of the inflammatory marker CRP (C-reactive protein) have been suggested as a proxy for EC dysfunction, due to the proposed deleterious effects on the production of the vasoprotective agent nitric oxide (NO),^3,17,18^ although other studies dispute this.^19^ Plasma asymmetrical dimethylarginine, symmetrical dimethylarginine, and homoarginine (agents involved in NO synthesis) can be modified by exposure to CVD risk factors and have also been measured to infer EC status.^20–23^ Other inflammation-related markers, such as interleukin-6, ICAM-1 (intercellular adhesion molecule-1), VCAM1 (vascular cell adhesion molecule-1), E-selectin, P-selectin, and VWF (von Willebrand factor) have been used as markers for EC dysfunction.^23–26^ Plasma levels of these markers can be increased in response to CVD risk factor exposure,^27–29^ and higher E- and P-selectin levels were associated with impaired acetylcholine-dependent EC vasodilation in humans,^30^ indicating that they can be linked to vascular changes beyond the inflammatory response. It remains unclear whether EC dysfunction is synonymous with, or limited to, inhibited vasoreactivity or inflammation-induced activation. Furthermore, of the aforementioned markers, only E-selectin and VCAM1 have high EC specificity (and then only under conditions of inflammation); other markers used can originate from different cell types, for example, plasma P-selectin is predominantly from platelets, rather than EC,^31^ making interpretation in the context of vascular health more complex. Sex is known to have an important influence on CVD development^32^; risk factors, incidence, age of occurrence, severity, clinical presentation, and treatment response varies between males and females.^33–35^ Despite this, sex-specific biomarkers for vascular dysfunction are lacking. Thus, the scope and specificity of the current measurements of EC dysfunction are limited,^36^ and we lack clinical tools for risk profiling and monitoring of response following intervention to improve EC function.

Here, we used affinity proteomics to measure levels of 216 plasma proteins, which we previously predicted as having an EC-enriched expression profile across human vascular beds,^37^ in samples collected as part of the population-based study, the SCAPIS (Swedish Cardiopulmonary Bioimage Study) pilot (N=1005).^38^ We identified 38 proteins in plasma that were associated with exposure to CVD risk factors. Nine proteins were associated with ≥3 risk factors, including VWF, ERG (ETS transcription factor ERG), and HEG1 (heart development protein with EGF like domains 1). Sex-specific analysis revealed that female- or male-only associations could be observed between EC protein levels and risk factor exposure. Finally, we show a relationship between biomarker expression and CVD risk, determined by the Framingham risk score (FRS), presenting the concept of risk profiling through measurement of EC proteins in plasma.

## Methods

### Data Availability

Human umbilical vein EC (HUVEC) sequencing data is available on ArrayExpress (accession number E-MTAB-4897). Analysis results and median fluorescence intensity values are provided in Table I in the Data Supplement. Due to the nature of the sensitive personal data and study materials, the clinical data cannot be made freely available. However, by contacting the corresponding author (lynn.butler@ki.se) or study organization (www.scapis.org), procedures for sharing data, analytic methods, and study materials for reproducing the results or replicating the procedure can be arranged following Swedish legislation.

### Samples Analyzed Using Plasma Proteomics

Plasma samples were collected as part of the SCAPIS pilot.^38^ Participants were sampled at the Sahlgrenska University Hospital. Whole blood was collected in EDTA anticoagulant after an overnight fast and centrifuged at 2000*g* for 20 minutes. Plasma aliquots were snap-frozen and stored at −80 °C until usage. The original SCAPIS pilot cohort contained data from 1111 individuals; however, a total of 106 of these were excluded from this study, as either (1) there was no blood sample available (n=43) or (2) samples were preprocessing outliers, lacked some accompanying clinical information, or were in the underweight body mass index (BMI) group (n=63). Thus, a total of 1005 samples were analyzed. Risk factor exposure and laboratory parameters were measured as previously described^38^ (also see Table I, Tab_1 in the Data Supplement).

### Antibody Selection and Bead-Based Array Generation

EC candidate targets proteins were selected based on our previous studies, where we predicted transcripts enriched in EC across vascular beds.^37^ Polyclonal antibodies, targeting 216 of these proteins, were obtained from the Human Protein Atlas project resource (www.proteinatlas.org). Plasma protein profiles were generated using affinity proteomics, as described in detail previously.^39,40^ In brief, each antibody was coupled to a unique identity color-coded magnetic beads (1.76 µg/mL, MagPlex-C, Luminex Corp). Rabbit anti-human albumin (Dako) and donkey anti-human IgG (Jackson ImmunoResearch Laboratories) antibodies were used as controls for sample and rabbit IgG (Jackson ImmunoResearch Laboratories) and bare beads served as negative controls. Antibody-bead coupling was confirmed by R-phycoerythrin–conjugated donkey anti-rabbit IgG (Jackson ImmnoResearch) before suspension bead array generation.

### Plasma Labeling and Profiling

The procedure for plasma labeling and protein profiling was performed as described previously.^39,40^ Plasma samples for both cohorts were randomized according to age and sex and distributed into microtiter plates using a liquid handling device (Freedom EVO150, Tecan). Plasma samples were diluted 1:10 in PBS and labeled with NHS-PEO4-biotin (Pierce) by liquid handling transference (CyBi-SELMA, CyBio). Labeled samples were further diluted (1:50) in assay buffer, heat-treated at 56 °C for 30 minutes, cooled to room temperature, and then combined with the suspension bead array. Unbound proteins were removed by washing and proteins captured on the beads were detected through a R-phycoerythrin–conjugated streptavidin (Invitrogen), using Flexmap 3D instruments (Luminex Corp). Protein profiles were reported as median fluorescence intensity, corresponding to relative plasma levels of each protein candidate.

### Statistical Analysis

Median fluorescence Intensity obtained as readout on the FlexMap 3D instrument (Luminex Corp) was processed and visualized in the R statistical computing software (v 3.1.2 and 3.5.1, respectively) unless stated otherwise. A minimum of at least 32 beads per antibody/bead region was required for inclusion in the analysis. Outlier samples were identified by robust principal component analysis and excluded from further analysis.^41^ Median fluorescence intensity data were normalized by (1) probabilistic quotient normalization as accounting for any potential sample dilution effects^42^ and (2) multidimensional MA (M=log ratio; A=mean average, scales) normalization to minimize the difference amount the subgroups of the samples generated by experimental factor as multiple batches.^43^ Log-transformation was applied to reduce right-skewness in the proteomic data distribution. To identify differences in protein profiles and the association with CVD risk factors, we applied linear regression analysis for each antibody, adjusting for age and sex, to determine association with variable of interest (eg, BMI, hypertension, diabetes). Analysis was performed on the whole cohort (N=1005) and on sex-stratified subgroups ([female n=507, male n=498]). Protein candidates were denoted as associated with a CVD risk factor in all when (1) Bonferroni corrected (*P*<0.05/216=2.31×10^-^^4^) in the full analysis and (2) *P*<0.05 in both sex-stratified subgroups. Protein candidates were denoted as predominantly associated with a CVD risk factor in females or males when (1) sex-risk factor interaction was significant (*P*<0.05; see Table I, Tab_3, Table B in the Data Supplement), and (2) there was an association in one sex (*P*<0.01), and (3) there was no association with the same risk factor in the other sex (*P*>0.05; see Table I, Tab_3, Table A in the Data Supplement). All associations were tested using linear regression.

CVD risk factors were analyzed as continuous variables or categorized according to the Framingham study risk score tables. FRS for each subject was calculated based on the previously described formula,^44^ where the following information was required: age (years), sex (male/female), total cholesterol (CHO; mg/dL), HDL (high-density lipoprotein) cholesterol (mg/dL), current smoking (yes/no), antihypertensive treatment (yes/no), diabetes (yes/no), and physician-acquired (clinic) systolic blood pressure (mm Hg). Associations between individual proteins and FRS were determined by linear regression analysis. To investigate the relationship between FRS and plasma protein profile, model selection was performed by a bidirectional stepwise algorithm, based on the significance *P* values. Analysis was done in R, version 4.1.0 (R Core Team 2021). R: A language and environment for statistical computing. R Foundation for Statistical Computing, Vienna, Austria.) Model selection algorithm was done with the “losrr” package (Aravind Hebbali (2020). olsrr: Tools for Building OLS Regression Models. R package version 0.5.3.).

### Transcript Profiling: Isolated Human ECs

HUVEC sequencing data were generated as part of our previous publication^37^ and has been deposited in ArrayExpress (www.ebi.ac.uk/arrayexpress/) under accession number E-MTAB-4897. Briefly, HUVEC were isolated from umbilical cords from 4 different donors, as described.^45^ Cells were maintained in Medium 199 (M199, Invitrogen) containing 20% fetal calf serum, 28 μg/mL gentamycin, 2.5 μg/mL amphotericin B, 1 ng/mL epidermal growth factor, and 1 μg/mL hydrocortisone (all from Sigma) for 48 hours before processing. HUVEC cultures isolated using this method were 96% to 98% pure, determined by positive staining by flow cytometry of CD105, CD31, and VWF, and the expression of elevated levels of ICAM-1 and E-selectin following stimulation with the inflammatory cytokine interleukin-1β. Total HUVEC RNA was isolated using the RNeasy mini kit with QIAshredder (Qiagen) according to the manufacturer’s instructions. RNA integrity number was >8.0 for all samples. RNA sequencing (RNA-seq) was performed using the standard Illumina RNA-seq protocol. Fragments per kilobase of exon model per million mapped reads values were calculated using Cufflinks v2.1.2^46^ and Ensembl build 75.^47^ The number of protein-coding genes mapped was 20 073.

### Data Usage

Normalized microarray gene expression datasets for human bladder microvascular EC (GSM72644), human iliac artery EC (GSM72657, GSM72658, GSM72659, GSM72660), human saphenous vein EC (GSM72683, GSM72683), human umbilical artery EC (GSM72686, GSM72687, GSM72688, GSM72689, GSM72690, GSM72691), and human uterine microvascular EC (GSM72692, GSM72692) were derived from a public data set of 61 different normal human cell cultures (GSE3239, GE Codelink Human Uniset) downloaded from NCBI-GEO (www.ncbi.nlm.nih.gov/geo/).

The Genotype-Tissue Expression Project was supported by the Common Fund of the Office of the Director of the National Institutes of Health, and by NCI (National Cancer Institute), NHGRI (National Human Genome Research Institute), NHLBI (National Heart, Lung, and Blood Institute), NIDA (National Institute on Durg Abuse), NIMH (National Institute of Mental Health), and NINDS (National Institute of Neurological Disorders and Stroke). The data used for the analyses of transcript expression of candidate proteins in whole blood were obtained from the Genotype-Tissue Expression Portal (https://gtexportal.org/home/).

Data from human single-cell atlases^48,49^ and the Panglao database (https://panglaodb.se)^50^ were used to provide EC protein expression information in Table I, Tab 5 in the Data Supplement.

### Tissue Profiling: Human Tissue Sections

Tissue microarrays were generated and stained as part of our Human Protein Atlas project, as previously described.^51,52^ Briefly, formalin-fixed and paraffin-embedded tissue samples were sectioned, deparaffinized in xylene, hydrated in graded alcohols, and blocked for endogenous peroxidase in 0.3% hydrogen peroxide diluted in 95% ethanol. For antigen retrieval, a Decloaking chamber (Biocare Medical, CA) was used. Slides were boiled in Citrate buffer, pH6 (Lab Vision, CA). Primary antibody against CLDN5 (claudin 5; Thermofisher CAB002607), LAMC1 (laminin subunit gamma 1; Atlas Antibodies HPA001909) and COL15A1 (collagen type XV alpha 1 chain; Atlas Antibodies HPA017915), and a dextran polymer visualization system (UltraVision LP HRP polymer, Lab Vision) were incubated for 30 minutes each at room temperature, and slides were developed for 10 minutes using Diaminobenzidine (Lab Vision) as the chromogen. Slides were counterstained in Mayers hematoxylin (Histolab) and scanned using Scanscope XT (Aperio).

## Results

To identify potential biomarkers for EC dysfunction, we generated a protein candidate list for screening, based on our previous work, where we identified 234 protein-coding transcripts with predicted EC-enriched expression across human organs (ie, with significantly higher specificity to EC, versus other cell types).^37^ Antibodies targeting 216 proteins encoded by these transcripts were selected from the Human Protein Atlas project (www.proteinatlas.org/) based on availability, concentration, and reliability score (Figure I and Table I, Tab_2 [column A–B] in the Data Supplement). We performed affinity proteomic screening of plasma samples collected as part of the population-based SCAPIS pilot, where subjects aged 50 to 64 years were randomly selected from the Swedish population register^38^ (N=1005 [female n=507, male n=498]; study population details Table I, Tab_1 in the Data Supplement). Five protein candidates were excluded from subsequent analysis as the reported data was affected by sample storage location, indicating a stability issue and hence reduced suitability as possible candidates for a routine diagnostic setting (Figure I and Table I, Tab_2 marked red in the Data Supplement).

### Candidate Proteins Are Associated With CVD Risk Factors

**Figure 1.**
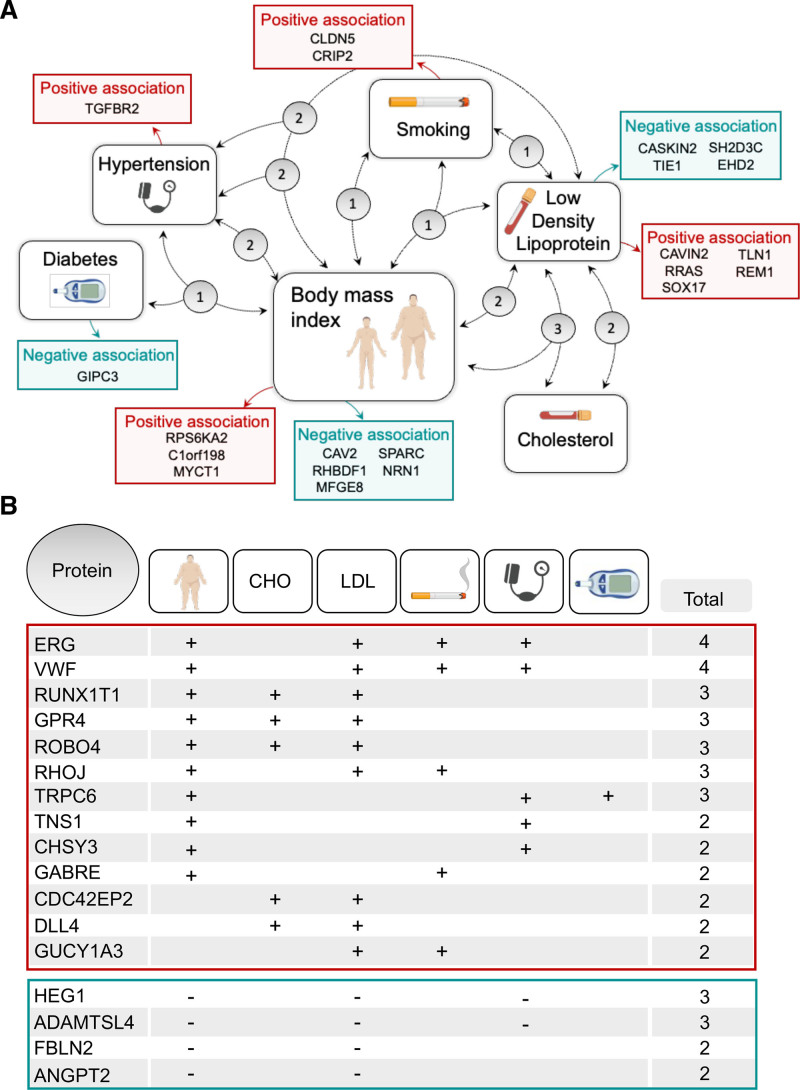
**Candidate plasma protein levels are associated with cardiovascular disease (CVD) risk factors.** Two hundred sixteen proteins with predicted endothelial cell (EC)-enriched expression were measured in plasma samples from male (n=498) and female (n=507) participants in the SCAPIS (Swedish Cardiopulmonary Bioimage Study) pilot. Candidates associated with the CVD risk factors body mass index (BMI), total cholesterol (CHO), LDL (low-density lipoprotein), smoking, hypertension, or diabetes were identified (age- and sex-adjusted linear model). **A**, Summary of EC-derived plasma proteins associated with CVD risk factors. Proteins associated with a single risk factor are displayed in adjacent red or green boxes, indicating a positive association (ie, higher protein levels associated with increased risk, for example, higher BMI, smoking) or a negative association (ie, lower protein levels associated with reduced risk profile, for example, lower BMI, lower blood LDL), respectively. Gray bubbles show the number of proteins associated with the (multiple) risk factors linked by the corresponding dotted line. **B**, Provides further details of these multiple risk factor–associated proteins, indicating if they are positively (+) or negatively associated (−) with the higher risk profile. ADAMTSL4 indicates ADAMTS-like 4; ANGPT2, angiopoietin 2; C1orf198, chromosome 1 open reading frame 198; CASKIN2, CASK-interacting protein 2; CAV2, caveolin 2; CAVIN2, caveolae-associated protein 2; CDC42EP2, CDC42 effector protein 2; CHSY3, chondroitin sulfate synthase 3; CLDN5, claudin 5; CRIP2, cysteine-rich protein 2; DLL4, delta-like canonical Notch ligand 4; EHD2, EH domain containing 2; ERG, ETS transcription factor ERG; FBLN2, fibulin 2; GABRE, gamma-aminobutyric acid type A receptor subunit epsilon; GIPC3, GIPC PDZ domain containing family member 3; GPR4, G-protein–coupled receptor 4; GUCY1A3, guanylate cyclase 1 soluble subunit alpha 3; HEG1, heart development protein with EGF-like domains 1; MFGE8, milk fat globule EGF and factor V/VIII domain containing; MYCT1, MYC target 1; NRN1, neuritin 1; REM1, RRAD and GEM–like GTPase 1; RHBDF1, rhomboid 5 homolog 1; RHOJ, ras homolog family member J; ROBO4, roundabout guidance receptor 4; RPS6KA2, ribosomal protein S6 kinase A2; RRAS, RAS related; RUNX1T1, RUNX1 partner transcriptional corepressor 1; SH2D3C, SH2 domain containing 3C; SOX17, SRY-box transcription factor 17; SPARC, secreted protein acidic and cysteine rich; TGFBR2, transforming growth factor beta receptor 2; TIE1, tyrosine kinase with immunoglobulin-like and EGF-like domains 1; TLN1, talin 1; TNS1, tensin 1; TRPC6, transient receptor potential cation channel subfamily C member 6; and VWF, von Willebrand factor.

We identified 38 candidate proteins (18% of all tested) that were associated with ≥1 of the following risk factors in both sexes: BMI, CHO, LDL (low-density lipoprotein), hypertension, smoking, or diabetes. A total of 21 proteins were associated with a single CVD risk factor (Figure 1A, red and green colored boxes represent positive and negative association, respectively, Table I Tab_2 in the Data Supplement), whereas 17 were associated with ≥2 risk factors (Figure 1A, gray circles show number of proteins linked to all connecting risk factors and Figure 1B). When associated with multiple risk factors, proteins were either consistently elevated with increased risk, for example, ERG (Figure 1B, red box); detected at higher levels with increasing BMI, LDL, smoking, and hypertension (+ sign), or consistently reduced with elevated risk, for example, HEG1 (Figure 1B, green box); detected at lower levels with increasing BMI, LDL, and hypertension (− sign).

Obesity is associated with increased risk of both arterial and venous CVD,^53,54^ with BMI being an independent predictor of disease occurrence, even after adjustment for other risk factors.^54,55^ We identified 22 EC proteins that were associated with BMI, making it the risk factor most frequently associated with modified levels of EC proteins in plasma (Figure 1A and 1B). Twenty out of 22 (91%) of the BMI-associated EC proteins in plasma were also associated with CRP, with the same effect direction (Table I, Tab_2 in the Data Supplement). CHO and diabetes were associated with plasma levels of 5 and 2 EC protein(s), respectively, representing the risk factors associated with the lowest number of proteins.

All candidates for screening were selected based on results from our previous study, where we used bioinformatic analysis of bulk RNA-seq data to predict which transcripts had EC-enriched expression across human organs.^37^ To verify the expression profile of the 38 candidate proteins identified as CVD risk factor associated, we collated RNA-seq data from the analysis of isolated EC,^37^ whole blood,^56^ or body-wide single-cell sequencing studies.^48–50^ The proteins were expressed at varying levels across human EC isolated from different vascular beds (Table I, Tab_5, Table A, column E, I, J, K, L, M in the Data Supplement), and although some transcripts were also detected in whole blood, levels were typically low (20/38 [53%]=0–1 TPM [transcripts per kilobase million]). Transcripts in blood could indicate expression by blood cells or could reflect the presence of circulating EC. In 2 body-wide human single-cell atlases^48,49^ and 1 single-cell database (of both mouse and human studies),^50^ the majority of the 38 candidates were enriched in EC; 20/38 (53%) in 2 or all, with 25/38 (66%) in at least 1 (Table I, Tab_5, Table A, column O, P, Q in the Data Supplement). Transcripts encoding for 13/38 candidates (34%) were enriched in immortalized EC, versus other cell lines (Table I, Tab_5, Table A, column S in the Data Supplement). In the peptide atlas,^57^ 22 out of 38 candidates (56%) had been previously detected in plasma with high confidence, using standard mass spectrometry (Table I, Tab_5, Table A, column W in the Data Supplement), which has lower sensitivity compared to the affinity-based protocol used in this study. A reported EC-specific functional role in the literature was found for 31 out of 38 candidates (82%; Table I, Tab_5, Table A, column AA and AC in the Data Supplement). Thus, there is supportive evidence that, as predicted in our original study, most candidate proteins are EC enriched. However, it should be acknowledged that, in some cases, other cell types likely contribute to plasma levels.

### Candidate Proteins Can Be Associated With Multiple Risk Factors

We identified a set of 17 EC proteins that were positively (n=13), or negatively (n=4) associated with ≥2 CVD risk factors, in both sexes (Figure 1B). There were 10 EC proteins associated with both BMI and LDL, making them the most common shared risk factor (Figure 1B). Consistent with previous reports,^58^ there was no correlation between LDL and BMI across the cohort (ρ<0.1), and adjustment for BMI did not affect any EC protein associations with LDL, or vice versa, further indicating that these risk factor associations were independent. Seven out of 22 (32%) of the BMI-associated proteins were also associated with hypertension. Elevated BMI is well known to be linked to hypertension,^59,60^ and the hypertension group (n=317), on average, had slightly higher BMI than the nonhypertension group (n=688; mean±SD: 26.5±4.0 versus 29.2±4.8 *P*<0.00001). For proteins associated with both, adjustment for the other factor reduced association strength, consistent with a probable interplay. EC protein associations with smoking were not modified by adjustment for any other risk factor.

**Figure 2.**
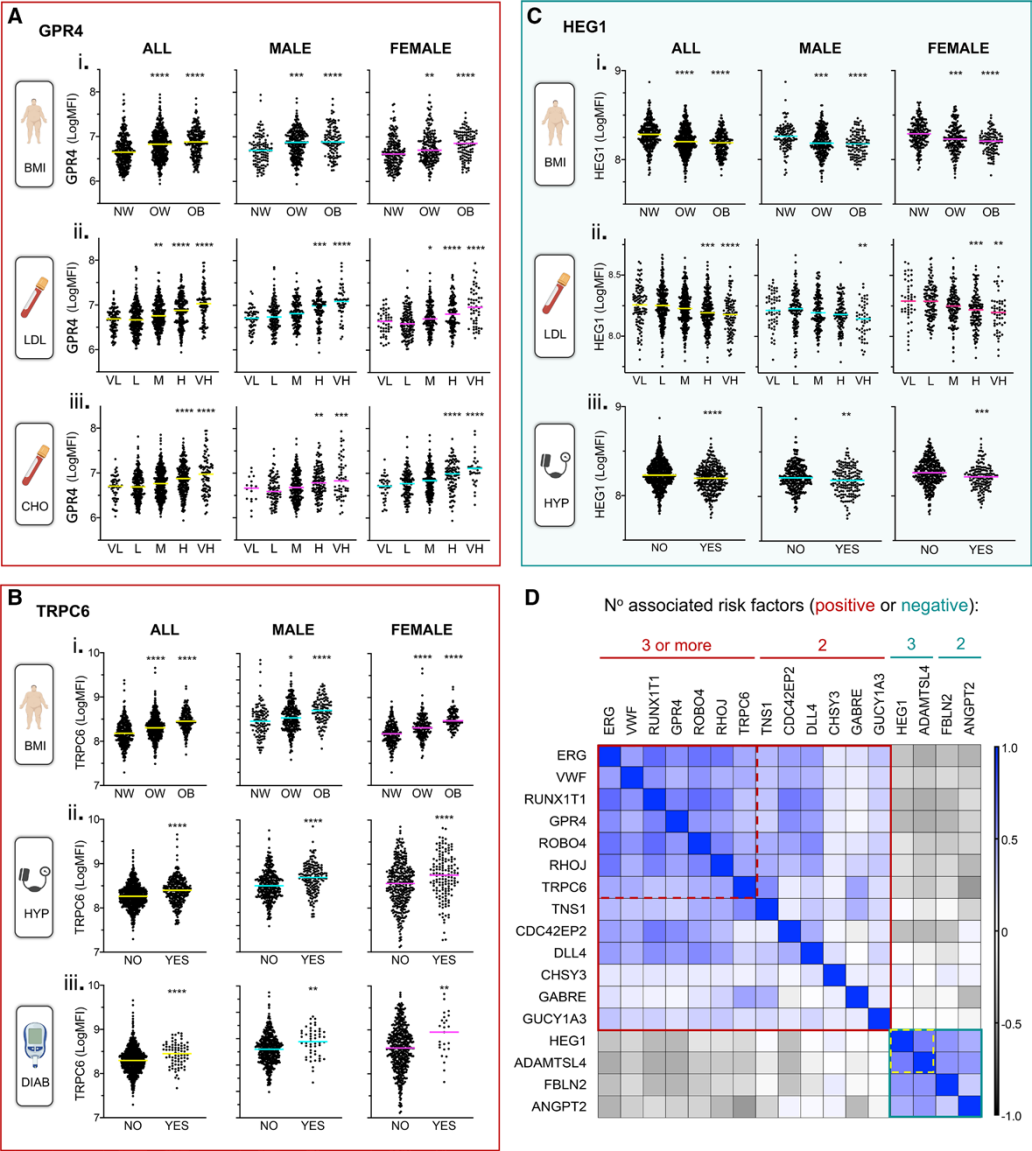
**Candidate plasma protein levels can be associated with multiple cardiovascular disease (CVD) risk factors.** Two hundred sixteen proteins with predicted endothelial cell (EC)–enriched expression were measured in plasma samples from male (n=498) and female (n=507) participants in the SCAPIS (Swedish Cardiopulmonary Bioimage Study) pilot (N=1005). Candidate proteins associated with CVD risk factors: body mass index (BMI), total cholesterol (CHO), LDL (low-density lipoprotein), hypertension (HYP), smoking, or diabetes (DIAB) were identified (age- and sex-adjusted linear model, [Bonferroni corrected *P* value]). Illustrative plots of relative plasma levels of proteins positively: (**A**) GPR4 (G-protein–coupled receptor 4; **B**) TRPC6 (transient receptor potential cation channel subfamily C), or negatively: (**C**) HEG1 (heart development protein with EGF like domains 1), associated with CVD risk factors, in (i) all, (ii) male or (iii) female-only samples. **D**, Heatmap matrix showing Spearman correlation coefficients between relative plasma levels of proteins associated with ≥2 risk factors across samples. Scale on right side of heatmap. *P* value *<0.05 **<0.01 ***<0.001 ****<0.0001 vs normal for BMI, and vs low for CHO and LDL (ANOVA followed by Tukey multiple comparisons, or unpaired *t* test [HYP, DIAB]). BMI: normal weight (NW: 18.5–24.9), overweight (OW: 25.0–29.9), obese (OB: >30). CHO: (mmol/L): very low (VL; <4.1), low (L: 4.1–5.1), moderate (M: 5.2–6.2), high (H: 6.3–7.2), very high (VH: ≥ 7.3). LDL: (mmol/L): very low (VL: <2.6), low (L: 2.6–3.4), moderate (M: 3.5–4.1), high (H: 4.2–4.9), very high (VH: ≥5). ADAMTSL4 indicates ADAMTS-like 4; ANGPT2, angiopoietin 2; CDC42EP2, CDC42 effector protein 2; CHSY3, chondroitin sulfate synthase 3; DLL4, delta-like canonical Notch ligand 4; ERG, ETS transcription factor ERG; FBLN2, fibulin 2; GABRE, gamma-aminobutyric acid type A receptor subunit epsilon; GPR4, G-protein -coupled receptor 4; GUCY1A3, guanylate cyclase 1 soluble subunit alpha 3; HEG1, heart development protein with EGF-like domains 1; MFI, median fluorescence intensity; RHOJ, ras homolog family member J; ROBO4, roundabout guidance receptor 4; RUNX1T1, RUNX1 partner transcriptional corepressor 1; TNS1, tensin 1; TRPC6, transient receptor potential cation channel subfamily C member 6; and VWF, von Willebrand factor.

Representative expression plots are shown for selected proteins associated with multiple risk factors (in the full cohort [All], male-only [male], and female-only [female] samples; Figure 2A through 2C). GPR4 (G-protein–coupled receptor 4) was positively associated with BMI (categorized into normal, overweight, or obese; Figure 2A, i), LDL (Figure 2A, ii), and CHO (Figure 2A, iii; both categorized as very low, low, moderate, high, or very high). TRPC6 (transient receptor potential cation channel subfamily C member 6) was also positively associated with BMI (Figure 2B, i), however, in contrast to GPR4, levels were not associated with LDL or CHO but were associated with hypertension (Figure 2B, ii) and diabetes (Figure 2B, iii). HEG1 was negatively associated with BMI (Figure 2C, i), LDL (Figure 2C, ii), and hypertension (Figure 2C, iii). Thus, risk factor associations with EC-derived protein level could be both protein- and risk factor-specific.

In all of these 17 cases, variation in protein levels across risk categories was similar between the full cohort, male-only, and female-only samples. However, 10 out of 13 (77%) of the EC proteins that were positively associated with multiple risk factors were present at significantly lower levels in female samples overall, compared to male samples (Figure IIA in the Data Supplement). Two of the 4 proteins that were negatively associated with risk factors were present at significantly higher levels in female samples, compared with male (Figure IIB in the Data Supplement). Thus, overall females had lower levels of risk-associated proteins and higher levels of those inversely correlated with elevated risk.

To determine if there was a potential relationship between risk-associated proteins within individuals, we calculated correlation coefficients (corr.) between protein levels across the full sample set. Proteins with the same effect direction, that is, positive or negatively associated with risk factors, generally correlated with each other across the cohort (Figure 2D [Figure IIIA in the Data Supplement shows all values]). The mean correlation was strongest between proteins associated with ≥3 risk factors (corr. ±SD, 0.50±0.12 [positive association], 0.62 [negative association] all *P*<0.00001; Figure 2D, indicated by red and yellow dashed lines, respectively). Positively associated proteins were generally modestly inversely correlated with negatively associated ones, the strongest inverse relationship observed between those associated with 3+ risk factors (mean corr. ±SD, −.27±0.09 *P*<0.00001). Equivalent analysis in male-only, or female-only, sample sets generated comparable results (Figure IIIB and IIIC in the Data Supplement). Thus, individuals with high plasma levels of any one of the multiple risk-associated EC proteins tended to have high levels of other such proteins, together with lower levels of (potentially) protective EC proteins. This is consistent with the concept that a plasma biomarker panel combination could together indicate degree of EC dysfunction on an individual level.

### Candidate Proteins Sex-Specifically Associated With CVD Risk Factors

**Figure 3.**
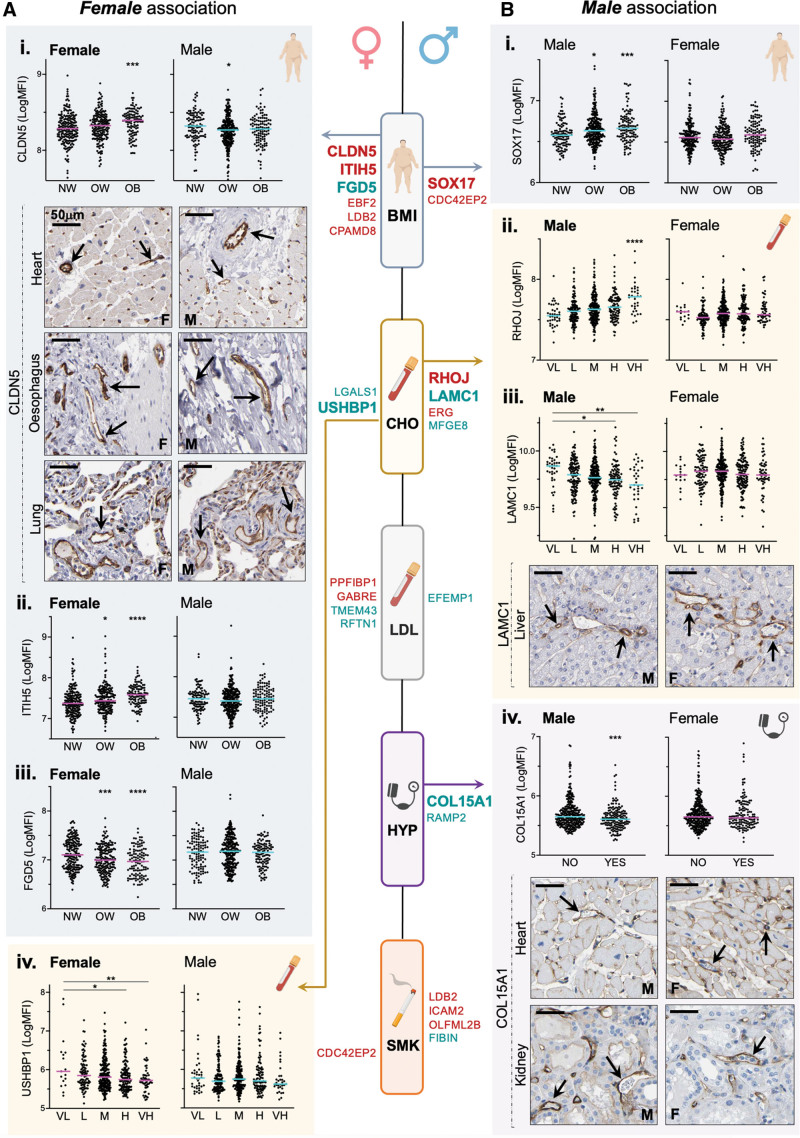
**Candidate plasma protein levels can be sex-specifically associated with cardiovascular disease risk factors.** Two hundred sixteen proteins with predicted endothelial cell (EC)–enriched expression were measured in plasma samples from male (n=498) or female (n=507) participants in the SCAPIS (Swedish Cardiopulmonary Bioimage Study) pilot. Candidates associated with body mass index (BMI), total cholesterol (CHO), LDL (low-density lipoprotein), hypertension (HYP), or smoking (SMK) predominantly in (**A**) females or (**B**) males were identified. Red or green text indicates a positive or negative association, respectively, between the protein levels and the annotated risk factor. Example plots show relative protein levels of candidates highlighted in large bold text. *<0.05 **<0.01 ***<0.001 ****<0.0001 vs normal for BMI, and vs low for CHO, unless otherwise indicated (ANOVA followed by Tukey multiple comparisons, or unpaired *t* test [HYP]). Female and male tissue sections from human heart, esophagus, lung, liver, or kidney were stained for CLDN5 (claudin 5), LAMC1 (laminin subunit gamma 1), or COL15A1 (collagen type XV alpha 1 chain); arrows indicate positive staining in blood vessels. BMI: normal weight (NW: 18.5–24.9), overweight (OW: 25.0–29.9), obese (OB: >30). CHO: (mmol/L): very low (VL;<4.1), low (L: 4.1–5.1), moderate (M: 5.2–6.2), high (H: 6.3–7.2), very high (VH: ≥ 7.3). LDL: (mmol/L): very low (VL: <2.6), low (L: 2.6–3.4), moderate (M: 3.5–4.1), high (H: 4.2–4.9), very high (VH: ≥5). CDC42EP2 indicates CDC42 effector protein 2; CPAMD8, C3 and PZP like alpha-2-macroglobulin domain containing 8; EBF2, EBF transcription factor 2; EFEMP1, EGF-containing fibulin extracellular matrix protein 1; ERG, ETS transcription factor ERG; F, female; FGD5, FYVE, RhoGEF and PH domain containing 5; FIBIN, fin bud initiation factor homolog; GABRE, gamma-aminobutyric acid type A receptor subunit epsilon; ICAM2, intercellular adhesion molecule 2; ITH5, inter-alpha-trypsin inhibitor heavy chain 5; LDB2, LIM domain-binding 2; LGALS1, galectin 1; M, male; MFGE8, milk fat globule EGF and factor V/VIII domain containing; MFI, median fluorescence intensity; OLFML2B, olfactomedin-like 2B; PPFIBP1, PPFIA-binding protein 1; RAMP2, receptor activity modifying protein 2; RFTN1, raftlin, lipid raft linker 1; RHOJ, ras homolog family member J; SOX17, SRY-box transcription factor 17; TMEM43, transmembrane protein 43; and USHBP1, USH1 protein network component harmonin binding protein 1.

There is relatively little known about sex-specific plasma protein profiles associated with CVD risk factors. We performed a sex-specific subgroup analysis (female n=507, male n=498, data for all proteins in Table I, Tab_3 and Tab_4 in the Data Supplement), which identified 26 candidates predominantly associated with specific CVD risk factor(s) in females or males (Figure 3 and Table I, Tab_4 in the Data Supplement; for details on classification, see methods). Thirteen proteins were associated with specific CVD risk factors in females, most frequently with BMI (6/13 [46%]); CLDN5 (Figure 3A, i), ITIH5 (inter-alpha-trypsin inhibitor heavy chain 5; Figure 3A, ii), FGD5 (FYVE, RhoGEF and PH domain containing 5; Figure 3A, iii), EBF2 (EBF transcription factor 2), LDB2 (LIM domain binging 2) and CPAMD8 (C3 and PZP like alpha-2-macroglobulin domain containing 8; Table I, Tab_4, Table A in the Data Supplement). Other female-only associations were observed between candidate proteins and CHO (Figure 3A, iv), LDL, and smoking (Figure 3 and Table I, Tab_4, Table A in the Data Supplement), but no associations were observed with hypertension. Thirteen proteins were associated with specific CVD risk factors in males only, most frequently with CHO or smoking (8/13 associations [62%]; Figure 3). Other male-only associations were observed between candidate proteins and BMI (Figure 3B, i), CHO (Figure 3B, ii and iii), hypertension (Figure 3B, iv), LDL, and smoking (Table I, Tab_4, Table B in the Data Supplement). Evidence for EC specificity of the sex-specifically associated candidates that were not associated with any other risk factor in the whole cohort is provided in Table I, Tab_5, Table 2 in the Data Supplement. In 3 body-wide human single-cell atlases,^48–50^ 8 out of 17 (47%) of these were EC-enriched in at least 1 dataset (Table I, Tab_5, Table B, column O, P, Q in the Data Supplement). Transcripts encoding for 6 out of 17 candidates (34%) were enriched in immortalized EC, versus other cell lines (Table I, Tab_5, Table B, column S in the Data Supplement), and 11 out of 17 candidates (65%) had a previously reported EC-specific functional role in the literature (Table I, Tab_5, Table B, column W in the Data Supplement). Thus, it should be acknowledged that other cell types also likely contribute to plasma levels of some of the described proteins. Immunohistochemistry staining showed vascular restricted expression of select candidate proteins in both female and male tissue from various organs (Figure 3A, i, Figure 3B, iii and iv). Sex-specific plasma protein profiles potentially indicate differing responses of the endothelium to CVD risk factor exposure in females and males.

### Candidate Protein Levels Are Associated With FRS

**Figure 4.**
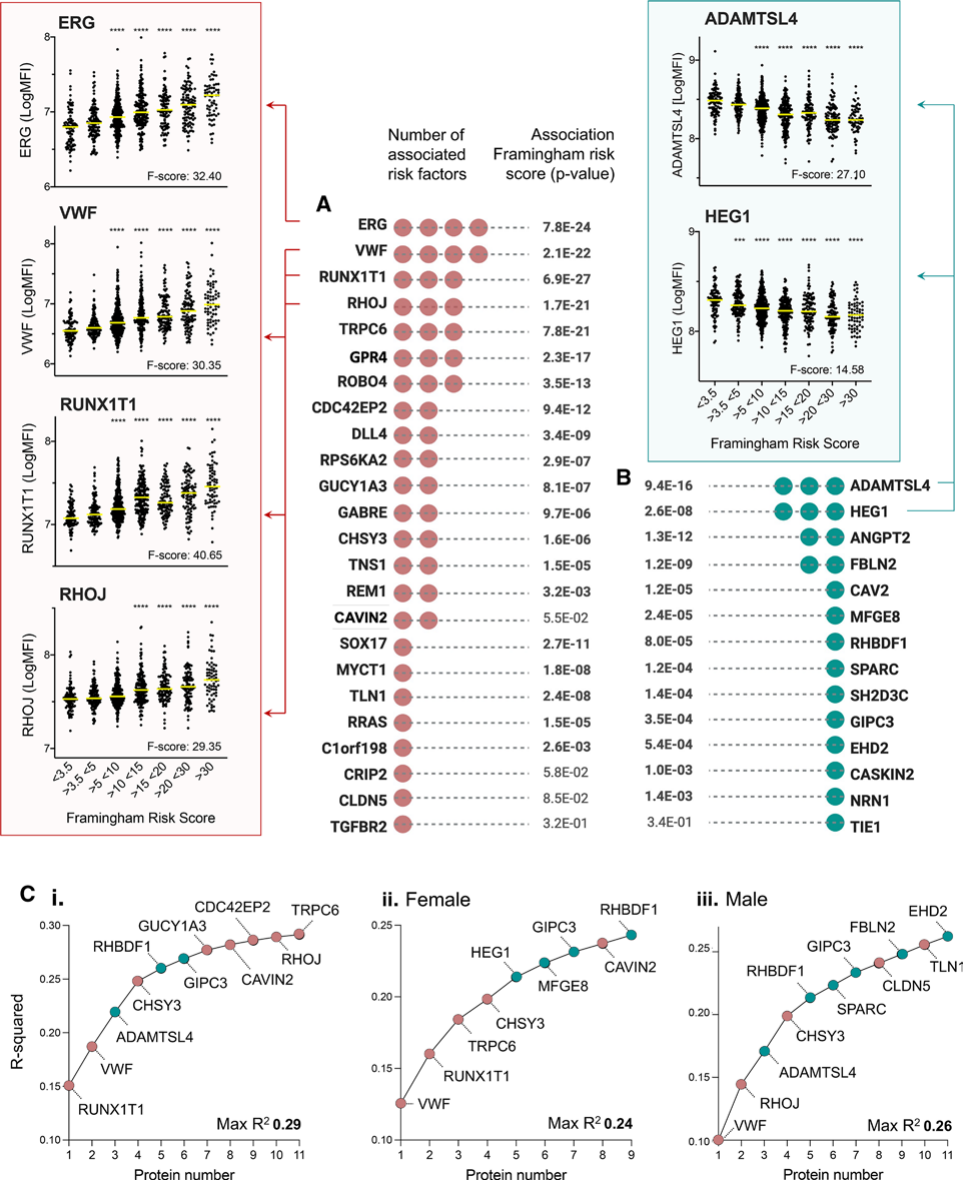
**Candidate plasma protein levels are associated with the Framingham risk score (FRS).** Two hundred sixteen proteins with predicted endothelial cell (EC)–enriched expression were measured in plasma samples from male and female participants in the SCAPIS (Swedish Cardiopulmonary Bioimage Study) pilot (N=1005). Candidates associated with body mass index, total cholesterol, LDL (low-density lipoprotein), smoking, hypertension, or diabetes were identified (age- and sex-adjusted linear model). FRS were calculated for each individual. Summary of EC proteins (**A**) positively or (**B**) negatively associated with ≥1 risk factor (adjacent red or green dots indicate number) and corresponding association between protein levels and the Framingham Risk scores across samples (linear model). Example plots show relative levels in plasma across the FRS groups for proteins positively (red box) or negatively (green box) associated with FRS. **C**, A bidirectional stepwise algorithm was used to identify EC proteins with an additive cumulative association with the FRS in (i) the whole cohort, or in (ii) female or (iii) male subgroup analysis. Direction of protein association with cardiovascular disease risk is indicated by color of point on the plot (red=positive, green=negative). *P* value ***<0.001 ****<0.0001 vs group >3.5 <5 (ANOVA [F score indicates variation between group means] and Tukey multiple comparisons). Framingham risk groups: <3.5 (n=94), >3.5 <5 (n=128), >5 <10 (n=303), >10 <15 (n=196), >15 <20 (n=107), >20 <30 (n=107), >30 (n=66). ADAMTSL4 indicates ADAMTS-like 4; ANGPT2, angiopoietin 2; C1orf198, chromosome 1 open reading frame 198; CASKIN2, CASK-interacting protein 2; CAV2, caveolin 2; CAVIN2, caveolae-associated protein 2; CDC42EP2, CDC42 effector protein 2; CHSY3, chondroitin sulfate synthase 3; CLDN5, claudin 5; CRIP2, cysteine-rich protein 2; DLL4, delta-like canonical Notch ligand 4; EHD2, EH domain containing 2; ERG, ETS transcription factor ERG; FBLN2, fibulin 2; GABRE, gamma-aminobutyric acid type A receptor subunit epsilon; GIPC3, GIPC PDZ domain containing family member 3; GPR4, G-protein–coupled receptor 4; GUCY1A3, guanylate cyclase 1 soluble subunit alpha 3; HEG1, heart development protein with EGF-like domains 1; MFGE8, milk fat globule EGF and factor V/VIII domain containing; MYCT1, MYC target 1; NRN1, neuritin 1; REM1, RRAD and GEM–like GTPase 1; RHBDF1, rhomboid 5 homolog 1; RHOJ, ras homolog family member J; ROBO4, roundabout guidance receptor 4; RPS6KA2, ribosomal protein S6 kinase A2; RRAS, RAS related; RUNX1T1, RUNX1 partner transcriptional corepressor 1; SH2D3C, SH2 domain containing 3C; SOX17, SRY-box transcription factor 17; SPARC, secreted protein acidic and cysteine rich; TGFBR2, transforming growth factor beta receptor 2; TIE1, tyrosine kinase with immunoglobulin-like and EGF-like domains 1; TLN1, talin 1; TNS1, tensin 1; TRPC6, transient receptor potential cation channel subfamily C member 6; and VWF, von Willebrand factor.

To determine if EC plasma proteins have potential utility in risk stratification, we calculated the FRS for each individual, as previously described^44^ (4 individuals were excluded as not all data was available), and measured the association with EC protein plasma levels (linear model). Thirty-three out of 38 (87%) of EC proteins that were associated with CVD risk factor(s) in the whole cohort analysis were associated with FRS (*P*<0.01; Figure 4A and 4B, *P* values annotated in bold). Of the proteins not associated with CVD risk factors in any analysis (ie, whole cohort, or single-sex), 127 out of 156 (82%) were not associated with the FRS. Relative expression of ERG, VWF, RUNX1T1 (RUNX1 partner transcriptional corepressor 1), and RHOJ (ras homolog family member J; Figure 4A, red box, higher risk associated) and ADAMTSL4 (ADAMTS-like 4) and HEG1 (Figure 4B green box, lower risk associated) are shown across groups with increasing FRS. ANOVA F scores (indicating the degree of variation between group means) are annotated on each plot and were consistent with the relative association *P* values. Thus, levels of EC proteins in the plasma reflect CVD risk, when measured using the FRS.

As we observed a relationship between positively and negatively CVD risk-associated EC proteins (Figure 2D), we investigated the concept of combining protein profiles to indicate relative risk. A bidirectional stepwise algorithm was used to determine if an additive cumulative association with the FRS was observed when multiple proteins were included in the model, based on their FRS association significance *P* values (Figure 4C). Whole cohort analysis revealed a strong cumulative effect for the first 4 proteins incorporated, which included those positively (RUNX1T1, VWF, and CHSY3 [chondroitin sulfate synthase 3]) and negatively (ADAMTSL4) associated with risk exposure (Figure 4C, i and Table I, Tab_6 in the Data Supplement). A weaker, but significant, effect was observed for each additional protein incorporated. The same analysis was performed using female (Figure 4C, ii) or male (Figure 4C, iii) sample subsets and, similar to the whole cohort analysis, a cumulative effect was observed with the addition of both positively and negatively risk-associated proteins.

Therefore, measurement of a panel of EC proteins in plasma more accurately reflects CVD risk, as measured by the FRS, than any individual protein.

## Discussion

Here, we identified a subset of EC expressed circulating proteins that are associated with exposure to CVD risk factors. Plasma levels of these proteins are associated with the FRS, providing a proof of concept that levels of EC expressed proteins found in plasma could reflect cardiovascular health. In a broader perspective, our study highlights the potential utilization of our ever-increasing knowledge of cell type–specific protein expression profiles for targeted biomarker exploration; prior knowledge that could facilitate functional investigations and, ultimately, pathophysiological understanding.

Accurate prediction of CVD risk is crucial when aiming for primary prevention therapies. The FRS is one of the most widely used prediction models, which is based on clinical parameters, but it lacks individualized precision. An individual´s susceptibility to CVD based on presence of environmental CVD risk factors is determined against a background of genetic disposition, including rare variants, and history of past exposure. However, risk-associated genetic variants contribute only marginally to CVD risk discrimination when incorporated into risk scores,^61,62^ possibly as the contribution to life-long risk exposure does not account for the modulating effects of nongenetic risk factor exposure that varies over time. From this perspective, plasma proteomics has the advantage of integrating both genetic and environmental influences, such as lifestyle changes and therapeutic interventions. In particular, interrogating the proteome of one of the main players of atherosclerotic development/susceptibility, the vascular wall, holds potential to identify novel markers that can provide a direct window into the current state of pathogenesis, and also indicate new biological pathways as novel targets for therapy. Conceptually, measurement of longitudinal EC proteins in plasma could provide a personalized assessment of vascular status over time, reflecting individual dynamic biological responses to dynamic changes in risk factor exposure, for example, weight loss or cessation of smoking. Our results could have further broad applicability beyond this, for example to monitor acute disease progression in conditions where EC function is central to disease pathology, such as coronavirus disease 2019 (COVID-19)^63,64^ or to monitor vascular response to drug treatment.

One strength of our study is the use of a population-based cohort design, covering a segment of the population aged 50 to 64 years, where CVD risk profiles may predispose to future events, but are difficult to stratify using current methods. All participants have been evaluated and sampled in a single location, by the same personnel and procedures, thereby controlling for most of postsampling factors that have been shown to potential bias or influence a proteomics-based study (needle-to-freeze-to-analysis).^65^ Another strength of our study is the targeted nature of the proteomic screening; expression specificity of a protein is a prerequisite for it to function as a useful biomarker of injury or disease of a particular tissue or cell type, for example, plasma levels of a cardiac-specific isoform of intracellular troponin (ie, TnT [troponin T]) are used to detect protein leakage from injured cardiomyocytes in myocardial infarction^66,67^ and plasma levels of prostate-specific antigen can be used to screen for prostate cancer.^68^ In both of the aforementioned cases, which represent some of the more commonly used clinical biomarkers today, the discovery as clinical biomarker followed the identification of the tissue and cell-specific expression patterns of the proteins. The target proteins we selected were based on our previous work where we predicted predominant expression in the EC compartment,^37^ providing highly relevant candidates to pursue as markers with potential specificity for vascular dysfunction. The Human Protein Atlas project^69^ allowed us full flexibility to design such a specific screening panel that, to our knowledge, constitutes the first large EC-centric plasma analysis. Identification of such positive and negative risk-associated proteins also generate new hypotheses and offer starting points for investigations into function or pathways that have a potential role in the pathophysiology associated with risk factor exposure.

Other screening technologies for CVD-relevant plasma proteins are available, such as commercial aptamer-based technology (www.somalogic.com) and proximity extension assays.^70^ Such assays have been used to identify biomarkers associated with CVD risk factor exposure, such as blood lipids and BMI,^71,72^ but these CVD screening panels are still primarily configured to detect proteins with known functions in pathophysiological processes and pathways involved in CVD (eg, inflammation, coagulation, lipid metabolism), and thus have limited overlap with our screening panel, which has a greater focus on biomarker source, rather than previous links to CVD. Furthermore, a significant number of candidates in such predeveloped screening panels have wide tissue or cell type expression, which can complicate interpretation of pathophysiological relevance of identified markers. Other existing proteomics methods, such as shot-gun mass spectrometry allow for global discovery, an unbiased or agnostic interrogation of the plasma proteome that can discover completely novel targets.^73^ However, these techniques have a lower overall sensitivity than the affinity proteomic approach used in our study, which may bias against the detection of biologically significant, but low abundant, proteins and give less accurate quantification.

With the exception of VWF, all of the proteins we identified as positively associated with multiple CVD risk factors are categorized as cell-associated, rather than secreted; GPR4, ROBO4 (roundabout guidance receptor 4), TRPC6, DLL4 (delta-like canonical Notch ligand 4), GABRE (gamma-aminobutyric acid type A receptor subunit epsilon) are membrane proteins,^74–78^ ERG and RUNX1T1 are transcription factors,^79,80^ RHOJ and TNS1 (tensin 1) are associated with focal adhesions,^81,82^ and CHSY3, CDC42EP2 (CDC42 effector protein 2) and GUCY1A3 (guanylate cyclase 1 soluble subunit alpha 3) are intracellular.^83–85^ CVD risk factor linked to elevated plasma levels of these proteins could reflect EC leakage, analogous to cardiac muscle troponin released following myocardial infarction. However, although the respective levels of some risk factor associated EC proteins correlate with each other across samples, others do not, indicating a level of complexity beyond that explained by a general leakage from the vasculature. The combination of risk factor exposures in any given individual, the vascular bed where the EC response or damage occurs, and the contribution from other pathways of release, such as via extracellular vesicles,^86^ could contribute to the specific plasma protein profile. Conversely, proteins negatively associated with multiple CVD risk factors, HEG1, ADAMTSL4, FBLN2 (fibulin 2), and ANGPT2 (angiopoietin 2) are all normally secreted.^87–90^ Other studies have examined plasma protein association with CVD risk factor exposure and prediction using predesigned proximity extension assays CVD panels; 102 proteins were identified as associated with baseline BMI in a study of weight loss over time, of which 88 were positively associated and 14 negatively associated,^71^ and 42 candidates were associated with at least one lipid fraction (triglycerides and total, LDL, or HDL cholesterol) with multiple proteins overlapping groups.^72^ In a study of 899 participants without overt CVD in the Framingham Heart Study Offspring cohort, 1129 proteins were measured, of which 156 were (positively or negatively) associated with FRS.^91^ However, other than VWF,^92^ to our knowledge there are no existing reports of the link between the proteins reported as associated in our study and CVD risk factor exposure or risk prediction, which could be explained by the limited cross over between our screening panel and those on commonly used preconfigured CVD screening panels.

There are limitations to our study. Although our antibodies passed quality control for antigen-binding specificity (see www.proteinatlas.org/), specificity for the target protein in context of the complex matrix of plasma still needs to be verified,^93^ to rule out nonspecific binding of other off-target candidates. The single binder assay format used, similar to other large-scale affinity proteomics assays, provides only a measure of relative quantification. Plasma levels of individual proteins cannot be directly compared to each other, as different antibodies have different binding affinities, and it is possibly for deviation from linearity to occur at the highest or lowest plasma concentrations. As a first step in developing standardized absolute quantification assays dual binder assays, such as ELISA, or antibody-free mass spectrometry-based approaches can be used for high throughput measurement of candidate proteins. Our results should then be replicated in a multicentre study. Our data could also be integrated with the genetic variation harbored by the participants to further pinpoint disease mechanism.^94^ The panel of proteins that we screened were originally predicted as being EC-enriched across tissue beds under normal conditions. Thus, it is likely that we have not measured EC biomarkers that are expressed only under conditions associated with CVD risk factor exposure, eg, inflammation. E-selectin is regulated by inflammation and is highly EC-specific, and the soluble versions of this protein have been identified as a potential marker for EC dysfunction and CVD.^95^ Although our knowledge of EC-specific gene expression under such conditions is expanding, it is currently limited and thus challenging to perform a comprehensive screen for such candidates. In addition, we cannot rule out that the expression of the identified biomarkers is induced in cell types other than EC, as a consequence of CVD risk factor exposure. We did not include tissue-specific EC-enriched candidates in our analysis panel, and thus we did not profile vascular bed-specific responses to CVD risk factor exposure. Such analysis could be of particular interest in the context of risk factors where the greatest effect could be anticipated to be manifested on a specific vascular bed, for example, the response of the lung vasculature to smoking exposure. Although we demonstrate a relationship between levels of EC expressed proteins found in plasma and FRS, candidate protein measurement in longitudinal samples, followed by association analysis with clinical outcomes, are needed to confirm the hypothesis that EC plasma protein risk profile and its variation over time could predict development of vascular-related diseases. In conclusion, through a targeted EC-centric analysis of plasma in relation to CVD risk factor exposure, we present the concept that EC protein profiles can reflect vascular health status.

## Article Information

### Acknowledgments

We would like to thank the team from the Translational Plasma Profiling Facility at SciLifeLab, Stockholm, for support and assistance in the generation of data for this project and the Biostatistics core facility at Karolinska Institute. Parts of some figures and the graphic abstract were created using BioRender.com.

### Sources of Funding

This work was supported by funding granted to L.M. Butler from Hjärt Lungfonden (20170759, 20170537) and the Swedish Research Council (2019-01493) and to J. Odeberg from Stockholm County Council (SLL/HMT, 2017-0842/0587), Familjen Erling Perssons Foundation, and HelseNord (HNF1544-20).

### Disclosures

None.

### Supplemental Materials

Data Supplement Figures I–III

Data Supplement Table I

## Supplementary Material


