# Supplementary material for: Identification of Endothelial Proteins in Plasma Associated With Cardiovascular Risk Factors
Source: Arterioscler Thromb Vasc Biol. 2021 Oct 28;41(12):2990–3004. doi: 10.1161/ATVBAHA.121.316779 (PMC8608011; doi:10.1161/ATVBAHA.121.316779)
Supplement: Supplementary file 1 [file atv-41-2990-s001.pdf]

SUPPLEMENTAL MATERIALS

Identification of endothelial proteins in plasma associated with cardiovascular risk factors

MJ Iglesias[1,2], LD Kruse[1], L Sanchez-Rivera[1], L Enge[1], P Dusart[1], MG Hong[1], M Uhlén[1], T Renné[3,4,5], JM Schwenk[1], G Bergstrom[6], J Odeberg[1,2,7,8], LM Butler [1,7,9*]

[1] Science for Life Laboratory, Department of Protein Science, CBH, KTH Royal Institute of Technology, SE-171 21 Stockholm, Sweden

[2] The University Hospital of North Norway (UNN), 9038 Tromsø, Norway

[3] Institute for Clinical Chemistry and Laboratory Medicine, University Medical Centre Hamburg-Eppendorf, D-20246 Hamburg, Germany

[4] Irish Centre for Vascular Biology, School of Pharmacy and Biomolecular Sciences, Royal College of Surgeons in Ireland, Dublin, Ireland.

[5] Centre for Thrombosis and Hemostasis (CTH), Johannes Gutenberg University Medical Center, Mainz, German

[6] Institute of Medicine, Sahlgrenska Academy at the University of Gothenburg, Gothenburg, Sweden

[7] Department of Clinical Medicine, The Arctic University of Norway, N-9037, Tromsø, Norway

[8] Coagulation Unit, Department of Hematology, Karolinska University Hospital, SE-171 76 Stockholm, Sweden

[9] Clinical Chemistry and Blood Coagulation Research, Department of Molecular Medicine and Surgery, Karolinska Institute, Stockholm, Sweden, *and* Clinical Chemistry, Karolinska University Laboratory, Karolinska University Hospital, Stockholm, Sweden

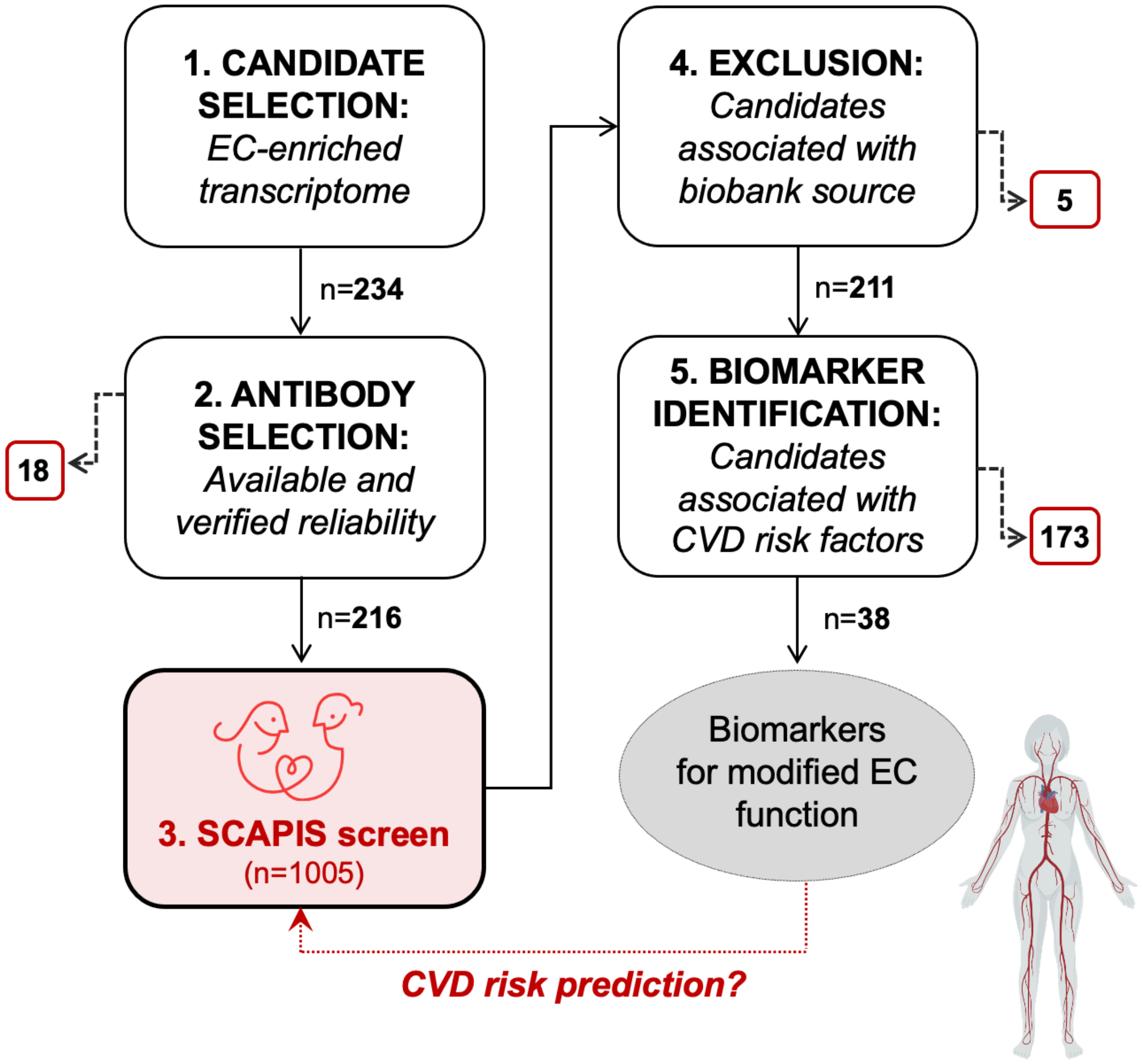

Figure SI. Affinity proteomic analysis workflow. (**1**) Candidate proteins for measurement in plasma were selected based on prior identification as predicted core endothelial (EC)-enriched genes across human tissue beds. (**2**) Antibodies targeting these candidate proteins were approved for screening (n=216), or not (n=18), based on availability and reliability (see methods). (**3**) Candidate proteins were measured in plasma samples (n=1005) collected as part of the *Swedish CArdioPulmonary bioImage Study* (SCAPIS) pilot. (**4**) If the protein profile was associated with biobank source, the data was excluded (n=5). (**5**) Protein profiles that were associated (age and sex adjusted linear model, [Bonferroni corrected; p=<2.31E-04]) with body mass index, smoking, diagnosis of hypertension, blood low density lipoprotein, blood cholesterol or diabetes were identified (n=38) and tested for association with the Framingham risk score.

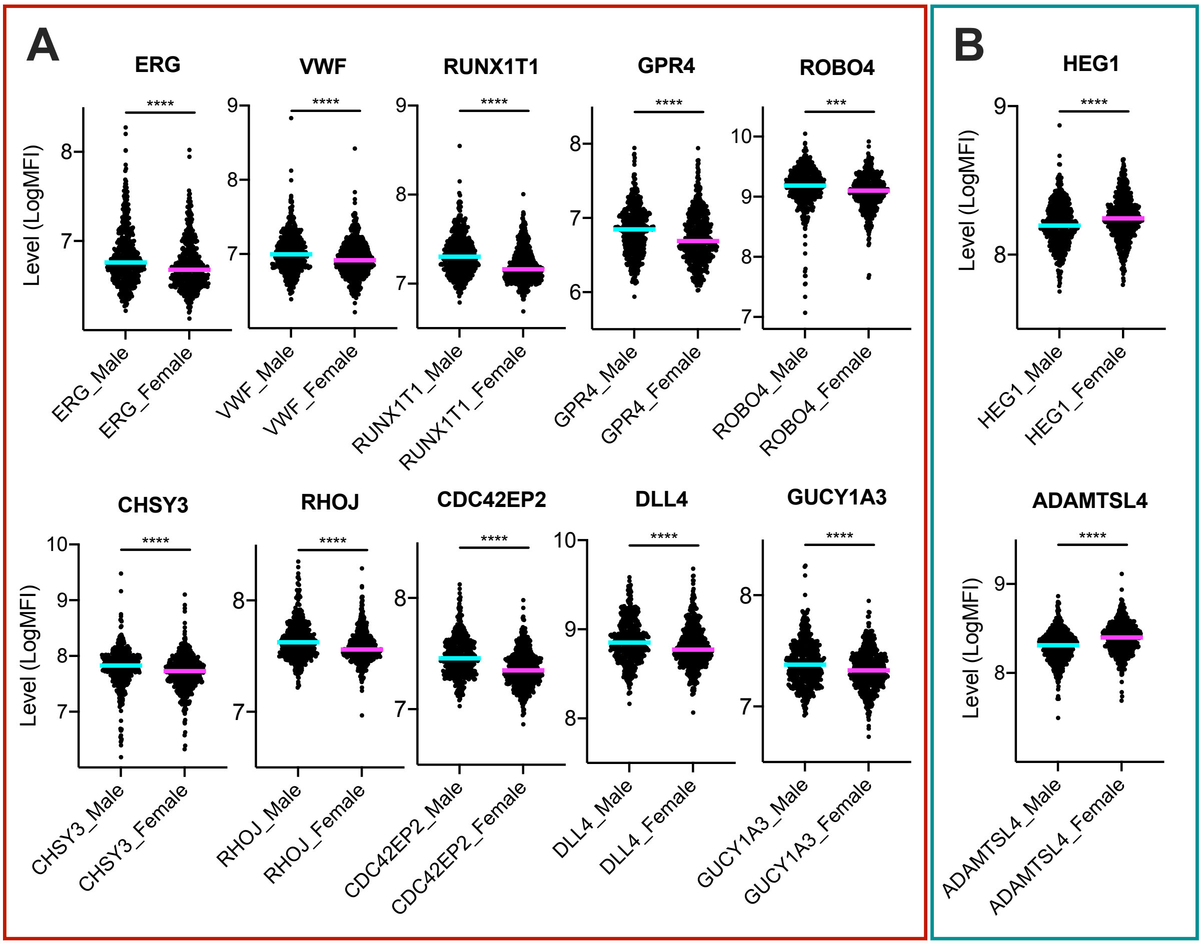

Figure SII. Plasma levels of endothelial proteins associated with multiple CVD risk factors, in male vs. female samples. Related to Figure 1 and 2. 216 proteins with predicted endothelial (EC)-enriched expression were measured in plasma samples from male (n=498) or female (n=507) participants in the *Swedish CArdioPulmonary bioImage Study* (SCAPIS) pilot. Relative plasma levels of proteins that were (**A**) positively (red box) or (**B**) negatively (green box) associated with multiple CVD risk factors in male and female samples. p-value *<0.05 **<0.01 ***<0.001 ****<0.0001 unpaired t-test.

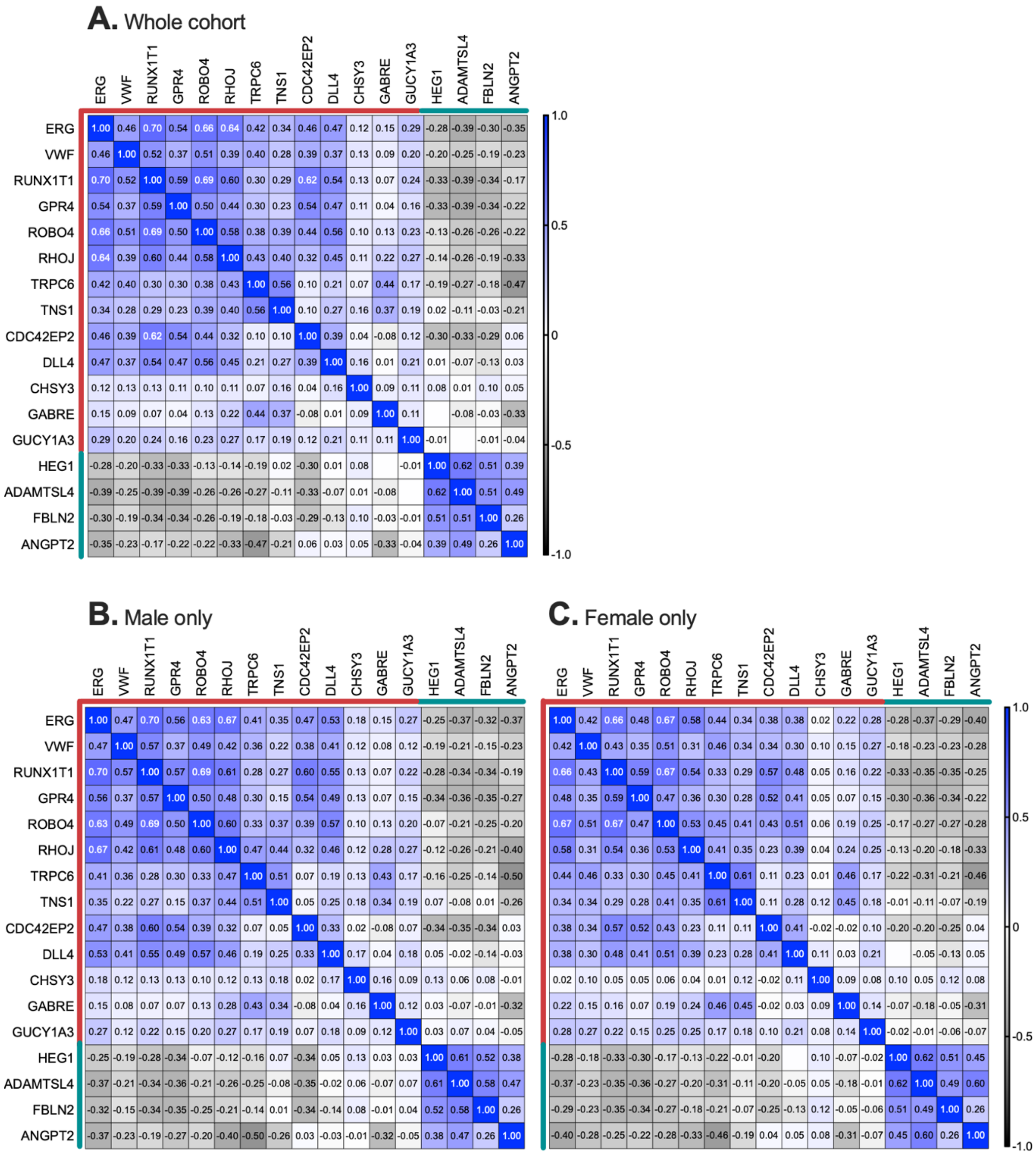

Figure SII. Correlation coefficients between plasma levels of endothelial proteins associated with multiple CVD risk factors. Related to Figure 2. 216 proteins with predicted endothelial (EC)-enriched expression were measured in plasma samples from male (n=498) and female (n=507) participants in the *Swedish CArdioPulmonary bioImage Study* (SCAPIS) pilot. Heatmaps show Spearman correlation coefficients between relative plasma levels of proteins positively or negatively (indicated by red or green line, respectively) associated with multiple CVD risk factors in: (**A**) the whole cohort, (**B**) male only or (**C**) female only samples.

DOI [to be added]

Major Resources Table

In order to allow validation and replication of experiments, all essential research materials listed in the Methods should be included in the Major Resources Table below. Authors are encouraged to use public repositories for protocols, data, code, and other materials and provide persistent identifiers and/or links to repositories when available. Authors may add or delete rows as needed.

Animals (in vivo studies)

Species	Vendor or Source	Background Strain	Sex	Persistent ID / URL
N/A				

Genetically Modified Animals

	Species	Vendor or Source	Background Strain	Other Information	Persistent ID / URL
**Parent - Male**	N/A				
**Parent - Female**	N/A				

Antibodies

Target antigen	Vendor or Source	Catalog #	Working concentration	Lot # (preferred but not required)	Persistent ID / URL
LGALS1	The Human Protein Atlas	**HPA000687**	17,6 µg/ml		https://www.atlasantibodies.com
A4GALT	The Human Protein Atlas	**HPA001141**	17,6 µg/ml		https://www.atlasantibodies.com
ELK3	The Human Protein Atlas	**HPA001600**	17,6 µg/ml		https://www.atlasantibodies.com
SEMA3G	The Human Protein Atlas	**HPA001761**	17,6 µg/ml		https://www.atlasantibodies.com
LAMB2	The Human Protein Atlas	**HPA001895**	17,6 µg/ml		https://www.atlasantibodies.com
LAMC1	The Human Protein Atlas	**HPA001908**	17,6 µg/ml		https://www.atlasantibodies.com
PPFIBP1	The Human Protein Atlas	**HPA001924**	17,6 µg/ml		https://www.atlasantibodies.com
FBLN2	The Human Protein Atlas	**HPA001934**	17,6 µg/ml		https://www.atlasantibodies.com
VWF	The Human Protein Atlas	**HPA002082**	17,6 µg/ml		https://www.atlasantibodies.com
FABP4	The Human Protein Atlas	**HPA002188**	17,6 µg/ml		https://www.atlasantibodies.com
CXorf36	The Human Protein Atlas	**HPA002806**	17,6 µg/ml		https://www.atlasantibodies.com
TM4SF1	The Human Protein Atlas	**HPA002823**	17,6 µg/ml		https://www.atlasantibodies.com
SPARC	The Human Protein Atlas	**HPA002989**	17,6 µg/ml		https://www.atlasantibodies.com
RHOJ	The Human Protein Atlas	**HPA003050**	17,6 µg/ml		https://www.atlasantibodies.com
LTBP2	The Human Protein Atlas	**HPA003415**	17,6 µg/ml		https://www.atlasantibodies.com

DOI [to be added]

SHANK3	The Human Protein Atlas	**HPA003446**	17,6 µg/ml		https://www.atlasantibodies.com
PTPRM	The Human Protein Atlas	**HPA003891**	17,6 µg/ml		https://www.atlasantibodies.com
LAMB1	The Human Protein Atlas	**HPA004132**	17,6 µg/ml		https://www.atlasantibodies.com
PECAM1	The Human Protein Atlas	**HPA004690**	17,6 µg/ml		https://www.atlasantibodies.com
TLN1	The Human Protein Atlas	**HPA004748**	17,6 µg/ml		https://www.atlasantibodies.com
C1orf198	The Human Protein Atlas	**HPA004798**	17,6 µg/ml		https://www.atlasantibodies.com
BCAM	The Human Protein Atlas	**HPA005654**	17,6 µg/ml		https://www.atlasantibodies.com
ADAMTSL4	The Human Protein Atlas	**HPA006279**	17,6 µg/ml		https://www.atlasantibodies.com
ACVRL1	The Human Protein Atlas	**HPA007041**	17,6 µg/ml		https://www.atlasantibodies.com
TBX2	The Human Protein Atlas	**HPA008586**	17,6 µg/ml		https://www.atlasantibodies.com
MCAM	The Human Protein Atlas	**HPA008848**	17,6 µg/ml		https://www.atlasantibodies.com
SOX7	The Human Protein Atlas	**HPA009065**	17,6 µg/ml		https://www.atlasantibodies.com
CD93	The Human Protein Atlas	**HPA009300**	17,6 µg/ml		https://www.atlasantibodies.com
HEG1	The Human Protein Atlas	**HPA010952**	17,6 µg/ml		https://www.atlasantibodies.com
TMEM109	The Human Protein Atlas	**HPA011785**	17,6 µg/ml		https://www.atlasantibodies.com
THSD1	The Human Protein Atlas	**HPA012611**	17,6 µg/ml		https://www.atlasantibodies.com
HSPA12B	The Human Protein Atlas	**HPA013659**	17,6 µg/ml		https://www.atlasantibodies.com
CYTH3	The Human Protein Atlas	**HPA013979**	17,6 µg/ml		https://www.atlasantibodies.com
GIMAP8	The Human Protein Atlas	**HPA014474**	17,6 µg/ml		https://www.atlasantibodies.com
ESYT1	The Human Protein Atlas	**HPA016858**	17,6 µg/ml		https://www.atlasantibodies.com
FBN1	The Human Protein Atlas	**HPA017759**	17,6 µg/ml		https://www.atlasantibodies.com
COL15A1	The Human Protein Atlas	**HPA017913**	17,6 µg/ml		https://www.atlasantibodies.com
NAV1	The Human Protein Atlas	**HPA018127**	17,6 µg/ml		https://www.atlasantibodies.com
NEK7	The Human Protein Atlas	**HPA018193**	17,6 µg/ml		https://www.atlasantibodies.com
ANGPT1	The Human Protein Atlas	**HPA018793**	17,6 µg/ml		https://www.atlasantibodies.com

DOI [to be added]

FGD5	The Human Protein Atlas	**HPA019191**	17,6 µg/ml		https://www.atlasantibodies.com
S100A13	The Human Protein Atlas	**HPA019592**	17,6 µg/ml		https://www.atlasantibodies.com
OLFML2A	The Human Protein Atlas	**HPA021180**	17,6 µg/ml		https://www.atlasantibodies.com
DLL4	The Human Protein Atlas	**HPA023392**	17,6 µg/ml		https://www.atlasantibodies.com
APBB2	The Human Protein Atlas	**HPA023542**	17,6 µg/ml		https://www.atlasantibodies.com
LMCD1	The Human Protein Atlas	**HPA024059**	17,6 µg/ml		https://www.atlasantibodies.com
NES	The Human Protein Atlas	**HPA026111**	17,6 µg/ml		https://www.atlasantibodies.com
ARHGAP29	The Human Protein Atlas	**HPA026534**	17,6 µg/ml		https://www.atlasantibodies.com
ATOH8	The Human Protein Atlas	**HPA028406**	17,6 µg/ml		https://www.atlasantibodies.com
ADAMTS9	The Human Protein Atlas	**HPA028567**	17,6 µg/ml		https://www.atlasantibodies.com
JAM2	The Human Protein Atlas	**HPA028789**	17,6 µg/ml		https://www.atlasantibodies.com
SASH1	The Human Protein Atlas	**HPA029947**	17,6 µg/ml		https://www.atlasantibodies.com
NRP1	The Human Protein Atlas	**HPA030278**	17,6 µg/ml		https://www.atlasantibodies.com
NPR1	The Human Protein Atlas	**HPA031087**	17,6 µg/ml		https://www.atlasantibodies.com
EPAS1	The Human Protein Atlas	**HPA031200**	17,6 µg/ml		https://www.atlasantibodies.com
CPAMD8	The Human Protein Atlas	**HPA031328**	17,6 µg/ml		https://www.atlasantibodies.com
ACKR3	The Human Protein Atlas	**HPA032003**	17,6 µg/ml		https://www.atlasantibodies.com
RBMS3	The Human Protein Atlas	**HPA034719**	17,6 µg/ml		https://www.atlasantibodies.com
FAM26E	The Human Protein Atlas	**HPA034970**	17,6 µg/ml		https://www.atlasantibodies.com
SCARF2	The Human Protein Atlas	**HPA035079**	17,6 µg/ml		https://www.atlasantibodies.com
VAMP5	The Human Protein Atlas	**HPA035082**	17,6 µg/ml		https://www.atlasantibodies.com
ACOT9	The Human Protein Atlas	**HPA035533**	17,6 µg/ml		https://www.atlasantibodies.com
TNS1	The Human Protein Atlas	**HPA036089**	17,6 µg/ml		https://www.atlasantibodies.com
ARHGAP31	The Human Protein Atlas	**HPA036380**	17,6 µg/ml		https://www.atlasantibodies.com
CD34	The Human Protein Atlas	**HPA036723**	17,6 µg/ml		https://www.atlasantibodies.com

DOI [to be added]

ADPRH	The Human Protein Atlas	**HPA036961**	17,6 μg/ml		https://www.atlasantibodies.com
CDC42EP2	The Human Protein Atlas	**HPA038562**	17,6 μg/ml		https://www.atlasantibodies.com
STARD13	The Human Protein Atlas	**HPA039535**	17,6 μg/ml		https://www.atlasantibodies.com
RAPGEF3	The Human Protein Atlas	**HPA040365**	17,6 μg/ml		https://www.atlasantibodies.com
SLC12A4	The Human Protein Atlas	**HPA041138**	17,6 μg/ml		https://www.atlasantibodies.com
HIF3A	The Human Protein Atlas	**HPA041141**	17,6 μg/ml		https://www.atlasantibodies.com
CRIP2	The Human Protein Atlas	**HPA042664**	17,6 μg/ml		https://www.atlasantibodies.com
ADAMTS15	The Human Protein Atlas	**HPA042867**	17,6 μg/ml		https://www.atlasantibodies.com
ARAP3	The Human Protein Atlas	**HPA042887**	17,6 μg/ml		https://www.atlasantibodies.com
CCDC85A	The Human Protein Atlas	**HPA043106**	17,6 μg/ml		https://www.atlasantibodies.com
PLAC9	The Human Protein Atlas	**HPA043469**	17,6 μg/ml		https://www.atlasantibodies.com
CHSY3	The Human Protein Atlas	**HPA044612**	17,6 μg/ml		https://www.atlasantibodies.com
CAV2	The Human Protein Atlas	**HPA044810**	17,6 μg/ml		https://www.atlasantibodies.com
GNG11	The Human Protein Atlas	**HPA045255**	17,6 μg/ml		https://www.atlasantibodies.com
ADAMTS7	The Human Protein Atlas	**HPA045284**	17,6 μg/ml		https://www.atlasantibodies.com
PKN3	The Human Protein Atlas	**HPA045390**	17,6 μg/ml		https://www.atlasantibodies.com
ERG	The Human Protein Atlas	**HPA046598**	17,6 μg/ml		https://www.atlasantibodies.com
EHD2	The Human Protein Atlas	**HPA047394**	17,6 μg/ml		https://www.atlasantibodies.com
SH2D3C	The Human Protein Atlas	**HPA047586**	17,6 μg/ml		https://www.atlasantibodies.com
CLEC14A	The Human Protein Atlas	**HPA048119**	17,6 μg/ml		https://www.atlasantibodies.com
ADCY4	The Human Protein Atlas	**HPA048344**	17,6 μg/ml		https://www.atlasantibodies.com
TINAGL1	The Human Protein Atlas	**HPA048695**	17,6 μg/ml		https://www.atlasantibodies.com
BMPR2	The Human Protein Atlas	**HPA049014**	17,6 μg/ml		https://www.atlasantibodies.com
EML1	The Human Protein Atlas	**HPA049105**	17,6 μg/ml		https://www.atlasantibodies.com
DOCK6	The Human Protein Atlas	**HPA049424**	17,6 μg/ml		https://www.atlasantibodies.com

DOI [to be added]

REM1	The Human Protein Atlas	**HPA049821**	17,6 µg/ml		https://www.atlasantibodies.com
PTRF	The Human Protein Atlas	**HPA049838**	17,6 µg/ml		https://www.atlasantibodies.com
EGFL7	The Human Protein Atlas	**HPA050716**	17,6 µg/ml		https://www.atlasantibodies.com
GIMAP6	The Human Protein Atlas	**HPA050740**	17,6 µg/ml		https://www.atlasantibodies.com
ISLR	The Human Protein Atlas	**HPA050811**	17,6 µg/ml		https://www.atlasantibodies.com
ESAM	The Human Protein Atlas	**HPA051043**	17,6 µg/ml		https://www.atlasantibodies.com
KANK3	The Human Protein Atlas	**HPA051153**	17,6 µg/ml		https://www.atlasantibodies.com
MAP3K6	The Human Protein Atlas	**HPA051192**	17,6 µg/ml		https://www.atlasantibodies.com
CD248	The Human Protein Atlas	**HPA051856**	17,6 µg/ml		https://www.atlasantibodies.com
TMEM88	The Human Protein Atlas	**HPA052991**	17,6 µg/ml		https://www.atlasantibodies.com
MEOX2	The Human Protein Atlas	**HPA053793**	17,6 µg/ml		https://www.atlasantibodies.com
FKBP9	The Human Protein Atlas	**HPA055256**	17,6 µg/ml		https://www.atlasantibodies.com
GAS6	The Human Protein Atlas	**HPA056080**	17,6 µg/ml		https://www.atlasantibodies.com
LIMS2	The Human Protein Atlas	**HPA058340**	17,6 µg/ml		https://www.atlasantibodies.com
GUCY1A3	The Human Protein Atlas	**HPA058617**	17,6 µg/ml		https://www.atlasantibodies.com
RBMS2	The Human Protein Atlas	**HPA058784**	17,6 µg/ml		https://www.atlasantibodies.com
FAM162B	The Human Protein Atlas	**HPA060342**	17,6 µg/ml		https://www.atlasantibodies.com
PHLDB1	The Human Protein Atlas	**HPA061506**	17,6 µg/ml		https://www.atlasantibodies.com
RBMS1	The Human Protein Atlas	**HPA061791**	17,6 µg/ml		https://www.atlasantibodies.com
CAVIN2	The Human Protein Atlas	**HPA062122**	17,6 µg/ml		https://www.atlasantibodies.com
OLFML2B	The Human Protein Atlas	**HPA062739**	17,6 µg/ml		https://www.atlasantibodies.com
ECSCR	The Human Protein Atlas	**HPA063337**	17,6 µg/ml		https://www.atlasantibodies.com
GPIHBP1	The Human Protein Atlas	**HPA066302**	17,6 µg/ml		https://www.atlasantibodies.com
ENG	The Human Protein Atlas	**HPA067440**	17,6 µg/ml		https://www.atlasantibodies.com
CYYR1	The Human Protein Atlas	**HPA067685**	17,6 µg/ml		https://www.atlasantibodies.com

DOI [to be added]

PTPRB	The Human Protein Atlas	**HPA067868**	17,6 µg/ml		https://www.atlasantibodies.com
FLT4	The Human Protein Atlas	**HPA067906**	17,6 µg/ml		https://www.atlasantibodies.com
SOX17	The Human Protein Atlas	**HPA068399**	17,6 µg/ml		https://www.atlasantibodies.com
EFEMP1	The Human Protein Atlas	**HPA070841**	17,6 µg/ml		https://www.atlasantibodies.com
CCM2L	The Human Protein Atlas	**HPA071063**	17,6 µg/ml		https://www.atlasantibodies.com
TCF7L1	The Human Protein Atlas	**HPA071298**	17,6 µg/ml		https://www.atlasantibodies.com
TEK	The Human Protein Atlas	**HPA073265**	17,6 µg/ml		https://www.atlasantibodies.com
BCL6B	The Human Protein Atlas	**HPA075112**	17,6 µg/ml		https://www.atlasantibodies.com
CASKIN2	The Human Protein Atlas	**HPA075184**	17,6 µg/ml		https://www.atlasantibodies.com
PREX2	The Human Protein Atlas	**HPA075956**	17,6 µg/ml		https://www.atlasantibodies.com
RPS6KA2	The Human Protein Atlas	**HPA003519**	17,6 µg/ml		Internal project catalog. https://www.proteinatlas.org
CD300LG	The Human Protein Atlas	**HPA004131**	17,6 µg/ml		Internal project catalog. https://www.proteinatlas.org
VEGFC	The Human Protein Atlas	**HPA004138**	17,6 µg/ml		Internal project catalog. https://www.proteinatlas.org
CALCRL	The Human Protein Atlas	**HPA007586**	17,6 µg/ml		Internal project catalog. https://www.proteinatlas.org
ACVR1	The Human Protein Atlas	**HPA008014**	17,6 µg/ml		Internal project catalog. https://www.proteinatlas.org
KDR	The Human Protein Atlas	**HPA008024**	17,6 µg/ml		Internal project catalog. https://www.proteinatlas.org
TGFBR2	The Human Protein Atlas	**HPA008766**	17,6 µg/ml		Internal project catalog. https://www.proteinatlas.org
HIPK3	The Human Protein Atlas	**HPA009703**	17,6 µg/ml		Internal project catalog. https://www.proteinatlas.org
MFAP5	The Human Protein Atlas	**HPA010012**	17,6 µg/ml		Internal project catalog. https://www.proteinatlas.org
SHROOM4	The Human Protein Atlas	**HPA010089**	17,6 µg/ml		Internal project catalog. https://www.proteinatlas.org
PDGFB	The Human Protein Atlas	**HPA011325**	17,6 µg/ml		Internal project catalog. https://www.proteinatlas.org
FLT1	The Human Protein Atlas	**HPA012053**	17,6 µg/ml		Internal project catalog. https://www.proteinatlas.org
AFAP1L1	The Human Protein Atlas	**HPA012099**	17,6 µg/ml		Internal project´s catalog. https://www.proteinatlas.org
TMEM204	The Human Protein Atlas	**HPA013658**	17,6 µg/ml		Internal project catalog. https://www.proteinatlas.org
GJA4	The Human Protein Atlas	**HPA014515**	17,6 µg/ml		Internal project catalog. https://www.proteinatlas.org

DOI [to be added]

CALHM2	The Human Protein Atlas	**HPA014698**	17,6 µg/ml		Internal project catalog. https://www.proteinatlas.org
TSPAN4	The Human Protein Atlas	**HPA016034**	17,6 µg/ml		Internal project catalog. https://www.proteinatlas.org
GPR4	The Human Protein Atlas	**HPA019207**	17,6 µg/ml		Internal project catalog. https://www.proteinatlas.org
MYCT1	The Human Protein Atlas	**HPA021515**	17,6 µg/ml		Internal project catalog. https://www.proteinatlas.org
COX4I2	The Human Protein Atlas	**HPA029307**	17,6 µg/ml		Internal project catalog. https://www.proteinatlas.org
PEAR1	The Human Protein Atlas	**HPA035218**	17,6 µg/ml		Internal project catalog. https://www.proteinatlas.org
HYAL2	The Human Protein Atlas	**HPA036435**	17,6 µg/ml		Internal project catalog. https://www.proteinatlas.org
ITIH5	The Human Protein Atlas	**HPA038304**	17,6 µg/ml		Internal project catalog. https://www.proteinatlas.org
GABRE	The Human Protein Atlas	**HPA038824**	17,6 µg/ml		Internal project catalog. https://www.proteinatlas.org
NID2	The Human Protein Atlas	**HPA039762**	17,6 µg/ml		Internal project catalog. https://www.proteinatlas.org
USHBP1	The Human Protein Atlas	**HPA041881**	17,6 µg/ml		Internal project catalog. https://www.proteinatlas.org
PLXND1	The Human Protein Atlas	**HPA042314**	17,6 µg/ml		Internal project catalog. https://www.proteinatlas.org
FZD4	The Human Protein Atlas	**HPA042328**	17,6 µg/ml		Internal project catalog. https://www.proteinatlas.org
BTNL9	The Human Protein Atlas	**HPA043429**	17,6 µg/ml		Internal project catalog. https://www.proteinatlas.org
ARHGEF15	The Human Protein Atlas	**HPA044443**	17,6 µg/ml		Internal project catalog. https://www.proteinatlas.org
TRPC6	The Human Protein Atlas	**HPA045098**	17,6 µg/ml		Internal project catalog. https://www.proteinatlas.org
LDB2	The Human Protein Atlas	**HPA051826**	17,6 µg/ml		Internal project catalog. https://www.proteinatlas.org
TNS2	The Human Protein Atlas	**HPA052162**	17,6 µg/ml		Internal project catalog. https://www.proteinatlas.org
AAED1	The Human Protein Atlas	**HPA052200**	17,6 µg/ml		Internal project catalog. https://www.proteinatlas.org
STARD8	The Human Protein Atlas	**HPA052832**	17,6 µg/ml		Internal project catalog. https://www.proteinatlas.org
TMEM255B	The Human Protein Atlas	**HPA053447**	17,6 µg/ml		Internal project catalog. https://www.proteinatlas.org
PDGFRB	The Human Protein Atlas	**HPA053716**	17,6 µg/ml		Internal project catalog. https://www.proteinatlas.org
COL4A1	The Human Protein Atlas	**HPA054039**	17,6 µg/ml		Internal project catalog. https://www.proteinatlas.org
ADAMTS2	The Human Protein Atlas	**HPA054331**	17,6 µg/ml		Internal project catalog. https://www.proteinatlas.org
RFTN1	The Human Protein Atlas	**HPA054662**	17,6 µg/ml		Internal project catalog. https://www.proteinatlas.org

DOI [to be added]

CLEC1A	The Human Protein Atlas	**HPA056307**	17,6 µg/ml		Internal project catalog. https://www.proteinatlas.org
NRN1	The Human Protein Atlas	**HPA057481**	17,6 µg/ml		Internal project catalog. https://www.proteinatlas.org
TM4SF18	The Human Protein Atlas	**HPA058640**	17,6 µg/ml		Internal project catalog. https://www.proteinatlas.org
DOCK9	The Human Protein Atlas	**HPA058757**	17,6 µg/ml		Internal project catalog. https://www.proteinatlas.org
VEGFB	The Human Protein Atlas	**HPA059415**	17,6 µg/ml		Internal project catalog. https://www.proteinatlas.org
FIBIN	The Human Protein Atlas	**HPA061451**	17,6 µg/ml		Internal project catalog. https://www.proteinatlas.org
DLC1	The Human Protein Atlas	**HPA061655**	17,6 µg/ml		Internal project catalog. https://www.proteinatlas.org
KIAA0355	The Human Protein Atlas	**HPA061806**	17,6 µg/ml		Internal project catalog. https://www.proteinatlas.org
TMEM43	The Human Protein Atlas	**HPA061839**	17,6 µg/ml		Internal project catalog. https://www.proteinatlas.org
LHFP	The Human Protein Atlas	**HPA062786**	17,6 µg/ml		Internal project catalog. https://www.proteinatlas.org
S1PR1	The Human Protein Atlas	**HPA062977**	17,6 µg/ml		Internal project catalog. https://www.proteinatlas.org
TANC1	The Human Protein Atlas	**HPA063446**	17,6 µg/ml		Internal project catalog. https://www.proteinatlas.org
FAM65A	The Human Protein Atlas	**HPA063782**	17,6 µg/ml		Internal project catalog. https://www.proteinatlas.org
CHST14	The Human Protein Atlas	**HPA063980**	17,6 µg/ml		Internal project catalog. https://www.proteinatlas.org
SVEP1	The Human Protein Atlas	**HPA064300**	17,6 µg/ml		Internal project catalog. https://www.proteinatlas.org
SHE	The Human Protein Atlas	**HPA064392**	17,6 µg/ml		Internal project catalog. https://www.proteinatlas.org
AMOTL2	The Human Protein Atlas	**HPA064426**	17,6 µg/ml		Internal project catalog. https://www.proteinatlas.org
RAMP2	The Human Protein Atlas	**HPA064452**	17,6 µg/ml		Internal project catalog. https://www.proteinatlas.org
EBF2	The Human Protein Atlas	**HPA064920**	17,6 µg/ml		Internal project catalog. https://www.proteinatlas.org
SOX18	The Human Protein Atlas	**HPA065347**	17,6 µg/ml		Internal project catalog. https://www.proteinatlas.org
LRRC32	The Human Protein Atlas	**HPA065439**	17,6 µg/ml		Internal project catalog. https://www.proteinatlas.org
RASIP1	The Human Protein Atlas	**HPA065655**	17,6 µg/ml		Internal project catalog. https://www.proteinatlas.org
RHBDF1	The Human Protein Atlas	**HPA065741**	17,6 µg/ml		Internal project catalog. https://www.proteinatlas.org
RUNX1T1	The Human Protein Atlas	**HPA066761**	17,6 µg/ml		Internal project catalog. https://www.proteinatlas.org
TIE1	The Human Protein Atlas	**HPA067134**	17,6 µg/ml		Internal project catalog. https://www.proteinatlas.org

DOI [to be added]

NOTCH3	The Human Protein Atlas	**HPA067424**	17,6 µg/ml		Internal project catalog. https://www.proteinatlas.org
GIPC3	The Human Protein Atlas	**HPA067765**	17,6 µg/ml		Internal project catalog. https://www.proteinatlas.org
RRAS	The Human Protein Atlas	**HPA068276**	17,6 µg/ml		Internal project catalog. https://www.proteinatlas.org
TIMP3	The Human Protein Atlas	**HPA068391**	17,6 µg/ml		Internal project catalog. https://www.proteinatlas.org
PCOLCE	The Human Protein Atlas	**HPA068422**	17,6 µg/ml		Internal project catalog. https://www.proteinatlas.org
PDGFRL	The Human Protein Atlas	**HPA068729**	17,6 µg/ml		Internal project catalog. https://www.proteinatlas.org
CLDN5	The Human Protein Atlas	**HPA068777**	17,6 µg/ml		Internal project catalog. https://www.proteinatlas.org
ANGPT2	The Human Protein Atlas	**HPA068842**	17,6 µg/ml		Internal project catalog. https://www.proteinatlas.org
COL4A2	The Human Protein Atlas	**HPA069337**	17,6 µg/ml		Internal project catalog. https://www.proteinatlas.org
TNXB	The Human Protein Atlas	**HPA070466**	17,6 µg/ml		Internal project catalog. https://www.proteinatlas.org
ITGB5	The Human Protein Atlas	**HPA071083**	17,6 µg/ml		Internal project catalog. https://www.proteinatlas.org
ICAM2	The Human Protein Atlas	**HPA071155**	17,6 µg/ml		Internal project catalog. https://www.proteinatlas.org
SLIT3	The Human Protein Atlas	**HPA071157**	17,6 µg/ml		Internal project catalog. https://www.proteinatlas.org
GJA5	The Human Protein Atlas	**HPA071370**	17,6 µg/ml		Internal project catalog. https://www.proteinatlas.org
LAMA2	The Human Protein Atlas	**HPA071462**	17,6 µg/ml		Internal project catalog. https://www.proteinatlas.org
HSPG2	The Human Protein Atlas	**HPA072690**	17,6 µg/ml		Internal project catalog. https://www.proteinatlas.org
EMCN	The Human Protein Atlas	**HPA073251**	17,6 µg/ml		Internal project catalog. https://www.proteinatlas.org
ROBO4	The Human Protein Atlas	**HPA073901**	17,6 µg/ml		Internal project catalog. https://www.proteinatlas.org
ZNF366	The Human Protein Atlas	**HPA074074**	17,6 µg/ml		Internal project catalog. https://www.proteinatlas.org
MMRN2	The Human Protein Atlas	**HPA074635**	17,6 µg/ml		Internal project catalog. https://www.proteinatlas.org
ARHGEF17	The Human Protein Atlas	**HPA074658**	17,6 µg/ml		Internal project catalog. https://www.proteinatlas.org
KIAA1462	The Human Protein Atlas	**HPA074823**	17,6 µg/ml		Internal project catalog. https://www.proteinatlas.org
EFEMP2	The Human Protein Atlas	**HPA074874**	17,6 µg/ml		Internal project catalog. https://www.proteinatlas.org
CDH5	The Human Protein Atlas	**HPA075875**	17,6 µg/ml		Internal project catalog. https://www.proteinatlas.org
PHLDB2	The Human Protein Atlas	**HPA076294**	17,6 µg/ml		Internal project catalog. https://www.proteinatlas.org

DOI [to be added]

MFGE8	The Human Protein Atlas	**HPA077076**	17,6 μg/ml		Internal project catalog. https://www.proteinatlas.org

DNA/cDNA Clones

Clone Name	Sequence	Source / Repository	Persistent ID / URL
N/A			

Cultured Cells

Name	Vendor or Source	Sex (F, M, or unknown)	Persistent ID / URL
N/A			

Data & Code Availability

Description	Source / Repository	Persistent ID / URL
HUVEC sequencing data	ArrayExpress	Accession number E-MTAB-4897

Other

Description	Source / Repository	Persistent ID / URL
N/A		

DOI [to be added]
