# Supplementary figures and images for: Identification of Endothelial Proteins in Plasma Associated With Cardiovascular Risk Factors
Source: Arterioscler Thromb Vasc Biol. 2021 Oct 28;41(12):2990–3004. doi: 10.1161/ATVBAHA.121.316779 (PMC8608011; doi:10.1161/ATVBAHA.121.316779)

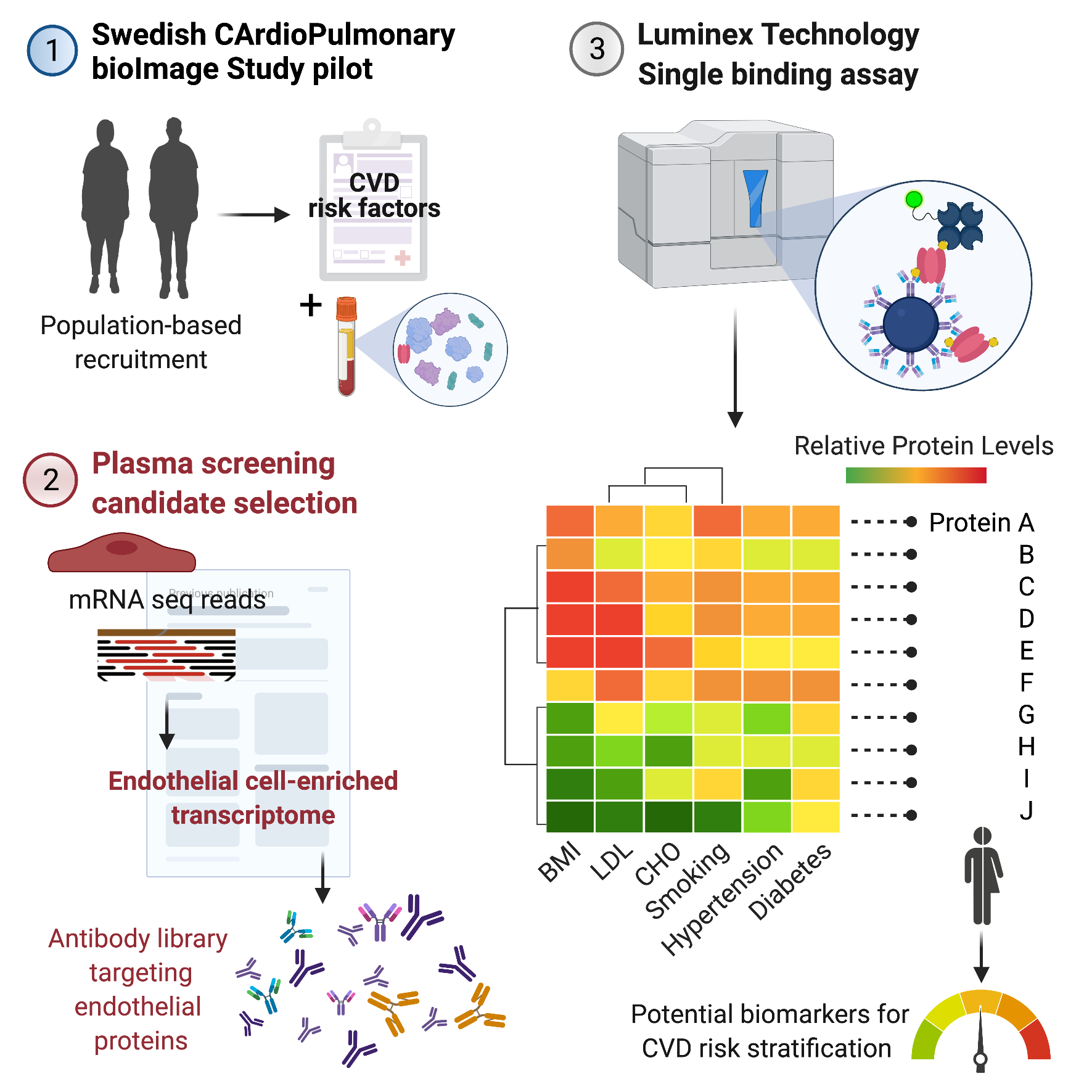

Supplement: Supplementary file 3 [file atv-41-2990-s003.jpg]
